# Comparison of innovative medical devices between China and the United States

**DOI:** 10.1093/rb/rbag008

**Published:** 2026-01-25

**Authors:** Xu Song, Bo Yuan, Kai Zhang, Chunying Chen, Yuliang Zhao, Antonios G Mikos, Xingdong Zhang

**Affiliations:** Institute of Regulatory Science for Medical Devices, Sichuan University, Chengdu 610064, China; School of Biomedical Engineering, Sichuan University, Chengdu 610064, China; National Engineering Research Center for Biomaterials, Sichuan University, Chengdu 610064, China; School of Biomedical Engineering, Sichuan University, Chengdu 610064, China; National Engineering Research Center for Biomaterials, Sichuan University, Chengdu 610064, China; Institute of Regulatory Science for Medical Devices, Sichuan University, Chengdu 610064, China; School of Biomedical Engineering, Sichuan University, Chengdu 610064, China; National Engineering Research Center for Biomaterials, Sichuan University, Chengdu 610064, China; CAS Key Laboratory for Biomedical Effects of Nanomaterials and Nanosafety and CAS Center for Excellence in Nanoscience, National Center for Nanoscience and Technology of China, Beijing 100190, China; College of Chemistry and Materials Science, Jinan University, Guangzhou 510632, China; CAS Key Laboratory for Biomedical Effects of Nanomaterials and Nanosafety and CAS Center for Excellence in Nanoscience, National Center for Nanoscience and Technology of China, Beijing 100190, China; College of Chemistry and Materials Science, Jinan University, Guangzhou 510632, China; Departments of Bioengineering, Chemical and Biomolecular Engineering, Rice University, Houston, TX 77251, USA; Institute of Regulatory Science for Medical Devices, Sichuan University, Chengdu 610064, China; School of Biomedical Engineering, Sichuan University, Chengdu 610064, China; National Engineering Research Center for Biomaterials, Sichuan University, Chengdu 610064, China

**Keywords:** innovative medical devices, breakthrough devices program, compound annual growth rate, IMD designations, marketing authorizations

## Abstract

From October 1, 2014 to June 30, 2025, China and the United States (U.S.) have implemented varying degrees of reform in the review and approval systems for innovative medical devices (IMDs), establishing and developing special review pathways for such devices. These reforms aim to further encourage the development of IMDs and accelerate the provision of safe and effective medical solutions to patients with unmet clinical needs. In this perspective, we analyze data on IMDs granted IMD Designations and that received marketing authorizations, and examine their development trends.

## Graphical Abstract

**Graphical Abstract:**
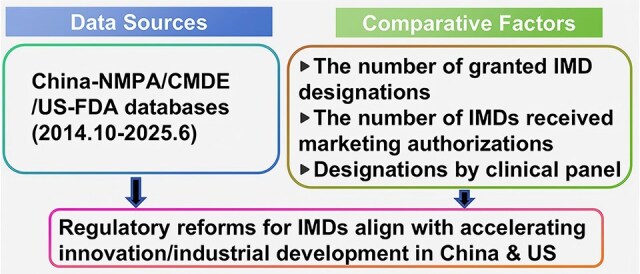

Unmet clinical needs and novel surgical approaches, biomaterials, and structural designs have driven the innovative development of medical devices. Although innovative medical devices (IMDs) offer unique benefits, their novelty and complexity present challenges for regulatory authorities, as well as increase the uncertainty and risks associated with the research, development, and commercialization of these products. From October 1, 2014 to June 30, 2025, China and the U.S. have established and developed special review pathways for such devices.

## IMD Policies in China and the U.S.

### China’s IMD policy

In 2014, the National Medical Products Administration (NMPA) of China issued the Trial Procedures for the Special Examination of Innovative Medical Devices (Special Procedures) [1]. In 2018, this was revised and reissued as the Special Approval Procedures for Innovative Medical Devices [2]. In 2025, Center for Medical Device Evaluation of NMPA issued Detailed Rules for the Review of Applications for Special Review of Innovative Medical Devices [3].

The aim of these policies is to ensure safety and effectiveness of IMDs, encourage research and innovation in the field of medical devices, promote the adoption and application of new medical technologies, and foster the development of the medical device industry.

### U.S. IMD policy

Since 1976, U.S. Food and Drug Administration (FDA) has been granted authority by Congress to regulate medical devices [4]. The 21st Century Cures Act provides $500 million in funding for FDA’s innovation projects, including reforms to the pre-market approval process for drugs and medical devices, aimed at accelerating review time while ensuring safety and effectiveness [5].

To prioritize the review of potentially IMDs, FDA established Priority Review Program (PRP) in 2013 [6]. However, since PRP was not sufficient to expedite the review process, FDA introduced Expedited Access Pathway (EAP) in 2015 to address these barriers. Under EAP, device manufacturers can communicate with FDA about pre-market approval [4]. In 2016, the U.S. Congress approved Breakthrough Devices Program (BDP), which replaced both PRP and EAP. BDP was updated in 2022 and officially revised in September 2023 to provide expedited review services for medical devices that treat life-threatening and irreversible degenerative diseases [6].

## Trends for IMDs in NMPA and FDA from October 1, 2014 to June 30, 2025 (the year begins on October 1 and concludes on September 30 of the subsequent year.) [7–12]

### The number of granted IMD designations shows an overall upward trend

**Figure 1:**
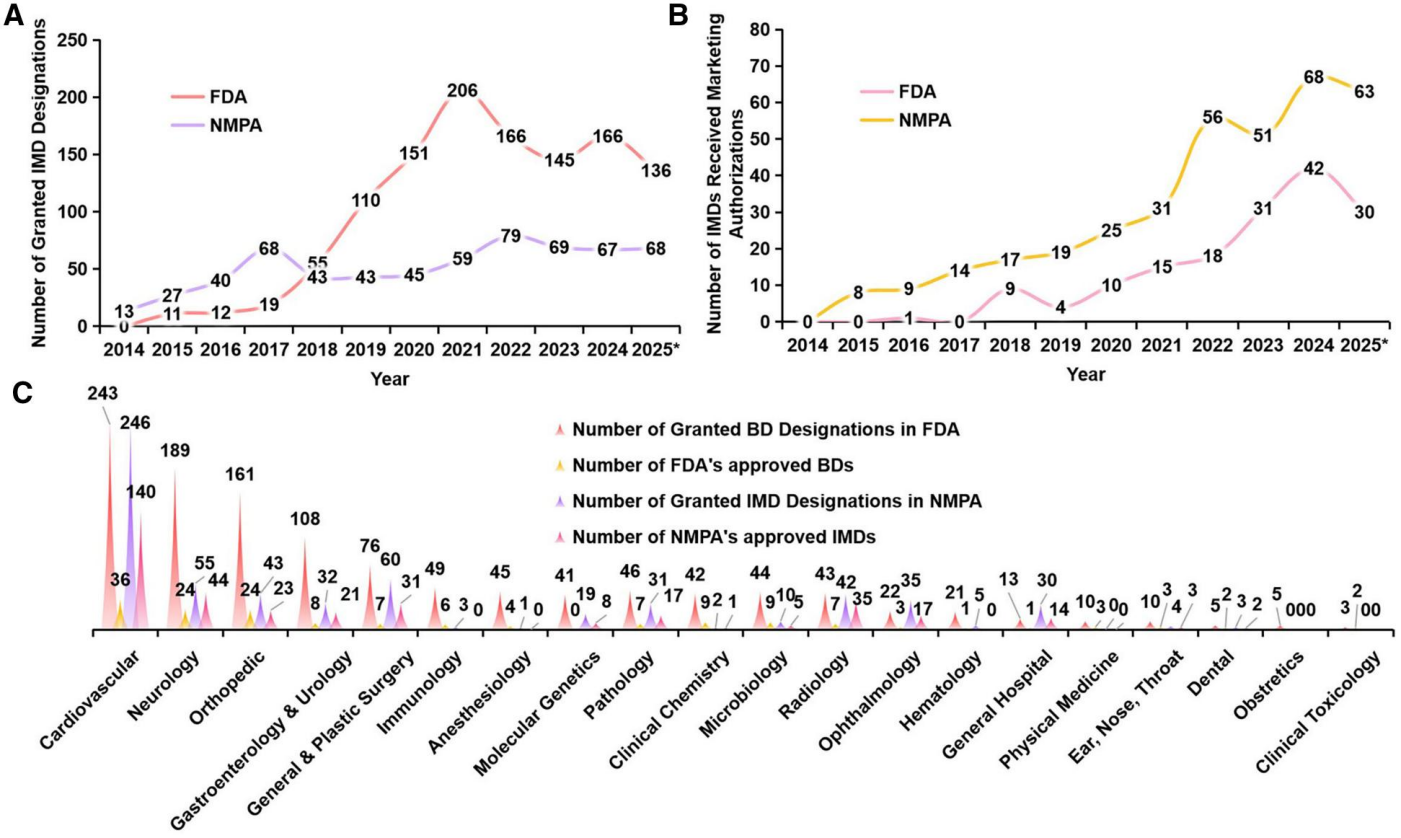
Trends for IMDs in NMPA and FDA from October 1, 2014 to June 30, 2025 [7–12]. (**A**). Annual number of granted IMD designations in NMPA and FDA. The FDA data for years 15–18 include devices designated under EAP. (**B**). Annual number of IMDs received marketing authorizations in NMPA and FDA. (**C**). Number of IMDs by clinical panel. *data only reflect from October 1, 2024 to June 30, 2025.

During the period from 2014 to 2024, the number of devices granted IMD designations in NMPA had a Compound Annual Growth Rate (CAGR) of 17.8% [13]; the number of devices granted Breakthrough Devices (BD) designations in FDA had a CAGR of 35.2%. (see the Figure 1A) Until June 30, 2025, the number of devices granted IMD designations in NMPA is 621, and that in FDA is 1177. The IMD policy can serve as a bridge from R&D to products and alleviate the backlog of applications. Although affected by market saturation, competitive environment and policies, the IMD policy still promotes the innovation of medical devices and maintains the fluctuating growth of the number of granted IMD designations.

### The number of IMDs received marketing authorizations is relatively low but is on the rise

As shown in Figure 1B, from 2015 to 2024, the CAGR for FDA approved IMDs is 55.7%, while NMPA’s CAGR is 24.5%. Until June 30, 2025, NMPA approved 361 IMDs, with an approval rate of 58.1%. During the same period, a total of 160 IMDs were approved in FDA, including 156 devices regulated by Center for Devices and Radiological Health (CDRH) and 4 devices regulated by Center for Biologics Evaluation and Research (CBER), with an approval rate of 13.6%. Both countries demonstrate sustained high growth rates, indicating that IMD policies are effectively promoting the timely market introduction of products that meet clinical needs.

### Granted IMD designations by clinical panel

As shown in Figure 1C, medical devices granted IMD designations in NMPA are concentrated in Cardiovascular (246, 39.6%), General & Plastic Surgery (60, 9.7%), Neurology(55, 8.9%), Orthopedic (43, 6.9%), and Radiology (42, 6.8%), while those granted BD designations in FDA is mainly concentrated in Cardiovascular (243, 20.6%), Neurology(189, 16.1%), Orthopedic (161, 13.7%), Gastroenterology & Urology (108, 9.2%), and General & Plastic Surgery (76, 6.8%). NMPA’s approved IMDs are mainly concentrated in Cardiovascular (140, 38.9%), Neurology (44, 12.2%), Radiology (35, 9.7%), General & Plastic Surgery (31, 8.6%), and Orthopedic (23, 6.4%), while FDA’s approved BDs are mainly concentrated in Cardiovascular (36, 22.5%), Neurology (24, 15%), Orthopedic (24, 15%), Microbiology (9, 5.6%) and Clinical Chemistry (9, 5.6%).

From October 1, 2014 to June 30, 2025, the regulatory reforms of medical devices in China and the U.S. have both reflected the goal of accelerating innovation and promoting industrial development. Although the specific measures and implementation methods of the two countries are different, their core concept is to break through the regulatory barriers of innovative products by optimizing the regulatory process, while ensuring the safety and effectiveness of the products. The IMD policy shortens the R&D and listing time of IMDs, saves the initial investment of medical device manufacturers, and helps patients and medical staff to obtain these medical devices in a timely manner to effectively treat and diagnose diseases.

Both China and the U.S. recognize that meeting clinical needs is an important factor in promoting medical device innovation. One of the recognition criteria for IMDs by NMPA and FDA is clinical demand, emphasizing that IMDs can provide breakthrough improvements in existing treatment methods, address unmet clinical needs and improve patient treatment outcomes, quality of life, and device safety. Both NMPA and FDA have published information on granted IMD Designations and IMDs received marketing authorizations, allowing medical staff and patients to obtain more accurate information for clinical decision-making.

Due to market demand, strong R&D capabilities of enterprises and policy support, the U.S. has led the development direction of IMDs and achieved a fast development of IMDs granted BD designations. At the same time, many of FDA-approved BDs are internationally first-in-class products. China’s medical device industry started later than that of the U.S. China has been continuously strengthening its policies to promote IMDs. Although the number of granted IMD designations in NMPA lags behind the U.S., it is in a relatively stable upward trend. The representative IMDs receiving marketing authorizations in NMPA and FDA are discussed in supplementary materials.

The application of new biomaterials, innovative structural designs, and/or innovations in surgical approaches has brought challenges to the supervision of IMDs. Although the review time for products granted IMD designations has been shortened, the lack of tools, standards and approaches to evaluate the safety and effectiveness of these products has led to a small number of approvals in NMPA or FDA. China’s advantage in the number of approved IMDs originates from initially leveraging international experience and obtaining approval for generic versions of international IMDs; however, China has gradually upgraded its products, and has approved internationally leading IMDs in various clinical panels.

Special Approval Procedures for Innovative Medical Devices in NMPA and BDP in FDA are new approaches of regulatory science [14]. New tools, standards and approaches of regulatory science, such as evidence-based research (including real-world research), computational modeling and simulation (CM&S), organ chips, and pre-review pathways, have actually promoted the launch of IMDs. Advancing regulatory science will improve the regulatory system for IMDs.

## Supplementary Material

rbag008_Supplementary_Data

## Data Availability

All data and materials generated or analyzed during this study are included in this published article (and its supplementary information files).
