# Supplementary material for: Comparison of innovative medical devices between China and the United States
Source: Regen Biomater. 2026 Jan 25;13:rbag008. doi: 10.1093/rb/rbag008 (PMC13003204; doi:10.1093/rb/rbag008)
Supplement: rbag008_Supplementary_Data [file rbag008_supplementary_data.zip › Table_S1.pdf]

Table S1. NMPA innovative medical devices Marketing Authorizations

NO.	Manufacturer	Trade Name	Registration Certificate Number	Registration Certificate Date(first approval)	Clinical Panel
1	Shenzhen Huayinkang Gene Technology Co., Ltd.	Gene Sequencer	National Medical Device Registration Approval No. 20143402171	12/10/2014	Molecular Genetics
2	Boao Biotechnology Co., Lt d.	RTisochip™-A: Isothermal Nucleic Acid Amplification Microfluidic Chip Analyzer	National Medical Device Registration Approval No. 20153400580	4/20/2015	Molecular Genetics
3	Suzhou Jingyu Medical De vices Co.,Ltd.	Dual channel implantable deep brain stimulation pulse generator kit	National Medical Device Registration Approval No. 20153210970	6/9/2015	Neurology
4	Suzhou Jingyu Medical De vices Co.,Ltd.	Implantable Deep Brain Stimulation Electrode Lead Kit	National Medical Device Registration Approval No. 20153210971	6/9/2015	Neurology
5	Suzhou Jingyu Medical De vices Co.,Ltd.	Implantable Deep Brain Stimulation Electrode Lead Kit	National Medical Device Registration Approval No. 20153210972	6/9/2015	Neurology
6	Xian GoldMag Nanobiotech Co., Ltd.	MTHFR C677T Gene Detection Kit (PCR-Gold Magnetic Particle Chromatography	National Medical Device Registration Approval No. 20153401148	7/3/2015	Molecular Genetics
7	Shenzhen AiNear Cornea Engineering Co., Ltd.	Acellular Corneal Stroma	National Medical Device Registration Approval No. 20153460581	4/22/2015	Ophthalmic
8	Boercheng (Beijing) Techn ology Co., Ltd.	Septin9 Gene Methylation Detection Kit (PCR fluorescent probe method)	National Medical Device Registration Approval No. 20153401481	8/24/2015	Pathology
9	Koning (Tianjin) Medical E quipment Co. LTD	Mammary X -ray Digital Tomography Equipment	National Medical Device Registration Approval No. 20153302052	11/20/2015	Radiology

10	Shanghai Wuseshi Medical Research Co. Ltd.	Survival Motor Neuron Gene 1 (SMN1) Exon Deletion Detection Kit (Fluorescence Quantitative PCR Method)	National Medical Device Registration Approval No. 20153402293	12/22/2015	Molecular Genetics
11	Shanghai MicroPort EP MedTech Co.,Ltd.	3D EP Navigation System	National Medical Device Registration Approval No. 20163770387	2/26/2016	Cardiovascular
12	Boao Biotechnology Co., Lt d.	Respiratory Tract Pathogenic Bacteria Nucleic Acid Detection Kit (Constant Temperature Amplification Chip Method)	National Medical Device Registration Approval No. 20163400327	2/17/2016	Microbiology
13	Guangzhou Youdi Bio-technology Co., Ltd.	Acellular Corneal Graft	National Medical Device Registration Approval No. 20163460573	3/28/2016	Ophthalmic
14	Beijing PINS Medical Co., Ltd.	Implantable Vagus Nerve Stimulation Pulse Generator Kit	National Medical Device Registration Approval No. 20163210989	5/16/2016	Neurology
15	Beijing PINS Medical Co., Ltd.	Implantable Vagus Nerve Stimulation Electrode Lead Kit	National Medical Device Registration Approval No. 20163210990	5/16/2016	Neurology
16	Beijing Xianruida Medical Technology Co., Ltd.	Drug-eluting Peripheral Balloon Dilatation Catheter	National Medical Device Registration Approval No. 20163771020	5/25/2016	Cardiovascular
17	Shanghai MicroPort EP MedTech Co.,Ltd.	Cold Saline Perfusion Radiofrequency Ablation Catheter	National Medical Device Registration Approval No. 20163771040	5/31/2016	Cardiovascular
18	Changzhou WastonOrtho Medical Appliance Co., Ltd.	Sternum Plate	National Medical Device Registration Approval No. 20163461582	10/8/2016	Orthopedic
19	MinFound Medical Systems Co., Ltd.	Positron Emission and Computed X-ray Tomography Imaging Device	National Medical Device Registration Approval No. 20163332156	10/17/2016	Radiology
20	Aibo Nord (Beijing) Medical Technology Co., Ltd.	Artificial Lens	National Medical Device Registration Approval No. 20163221747	11/21/2016	Ophthalmic

21	Beijing Tinavi Medical Technologies Co., Ltd.	Orthopedic Surgical Navigation Positioning System	National Medical Device Registration Approval No. 20163542280	2016.11.14	Orthopedic
22	Hygea Medical Technology Co., Ltd.	Cryoablation Cryoablation System	National Medical Device Registration Approval No. 20173583088	2/14/2017	General & Plastic Surgery
23	Hygea Medical Technology Co., Ltd.	Cryoablation Needles	National Medical Device Registration Approval No. 20173583089	2/14/2017	General & Plastic Surgery
24	Beijing Novel Medical Equipment Ltd.	Dual-Head Fully Digital General-Purpose Single-Photon Emission Computed Tomography Imaging Device	National Medical Device Registration Approval No. 20173330681	4/26/2017	Radiology
25	Puyi (Shanghai) Biotechnology Co., Ltd.	BISORB Drug-Eluting Sinus Absorbable System	National Medical Device Registration Approval No. 20173460679	4/25/2017	Ear, Nose, Throat
26	Hangzhou Qiming Medical Equipment Co., Ltd.	Percutaneous Interventional Prosthetic Heart Valve System	National Medical Device Registration Approval No. 20173460680	4/25/2017	Cardiovascular
27	Suzhou Jiecheng Medical Technology Co., Ltd.	Interventional Prosthetic Heart Valve System	National Medical Device Registration Approval No. 20173460698	4/28/2017	Cardiovascular
28	Beijing Yihe Medical Technology Co., Ltd.	Disposable Absorbable Nail Intradermal Stapler	National Medical Device Registration Approval No. 20173650874	5/31/2017	General & Plastic Surgery
29	Lifetech Scientific (Shenzhen) Co. Ltd.	Left Atrial Appendage Closure System	National Medical Device Registration Approval No. 20173770881	6/2/2017	Cardiovascular
30	MicroPort Scientific Corpor ation	Branched Aortic Stent-Graft System	National Medical Device Registration Approval No. 20173463241	6/26/2017	Cardiovascular
31	Guangzhou Weibo Biotechnology Co., Ltd.	Foldable Artificial Vitreous Body	National Medical Device Registration Approval No. 20173223296	7/25/2017	Ophthalmic

32	Beijing Percutek Therapeutics Medical Equipment Co., Ltd.	Abdominal Aortic Stent Graft System	National Medical Device Registration Approval No. 20173461434	10/17/2017	Cardiovascular
33	Lifetech Scientific (Shenzhen) Co. Ltd.	HeartTone™ Implantable Cardiac Pacemaker	National Medical Device Registration Approval No. 20173211570	12/11/2017	Cardiovascular
34	Amoy Diagnostics Co., Ltd.	Super-ARMS® EGFR Mutation Detection Kit	National Medical Device Registration Approval No. 20183400014	1/18/2018	Pathology
35	Shandong Saikesaisi Pharmaceutical Technology Co., Ltd.	Absorbable Dural Sealant	National Medical Device Registration Approval No. 20183650031	1/25/2018	General & Plastic Surgery
36	Shanghai Microport Medical (Group) Co., Ltd.	Flex Embolization Device	National Medical Device Registration Approval No. 20183770102	3/15/2018	Neurology
37	Shenzhen GeneBioHealth Co., Ltd.	miR-92a Detection Kit (Fluorescent RT-PCR)	National Medical Device Registration Approval No. 20183400108	3/27/2018	Pathology
38	Beijing NaGene Diagnosis Reagent Co., Ltd.	Hepatitis C Virus Nucleic Acid Assay Kit (PCR-Fluorescent Probe Method)	National Medical Device Registration Approval No. 20183400157	4/20/2018	Microbiology
39	Minitech Medical (Jiangsu) Ltd.	Cerebral Thrombosis Retrieval Device	National Medical Device Registration Approval No. 20183770186	5/8/2018	Neurology
40	Pulse Medical Technology, Inc.	Quantitative Flow Ratio Measurement System	National Medical Device Registration Approval No. 20183210282	7/12/2018	Radiology
41	Guangzhou Burning Rock Biotech Co., Ltd.	Human-targeted EGFR/ALK/BRAF/KRAS Polygene Mutation Testing Kit (Reversible	National Medical Device Registration Approval No. 20183400286	7/18/2018	Pathology
42	Beijing Unidiag Technology Inc.	UD90DT Automatic Electro-Chemiluminescence Immunoassay Analyzer	National Medical Device Registration Approval No. 20183220293	8/11/2018	General Hospital

43	Novogene Co, Ltd.	Human EGFR, KRAS, BRAF, PIK3CA, ALK, ROS1 Gene Mutation Detection Kit (Semiconductor Sequencing Method)	National Medical Device Registration Approval No. 20183400294	8/11/2018	Pathology
44	Shanghai Pine & Power Biotech Co., Ltd.	Composite Hernia Patch	National Medical Device Registration Approval No. 20183130292	8/12/2018	General & Plastic Surgery
45	Shanghai United Imaging Healthcare Co. Ltd.	Positron Emission Tomography and Magnetic Resonance Imaging System	National Medical Device Registration Approval No. 20183060337	8/29/2018	Radiology
46	Nanjing Shihe Medical Equipment Co., Ltd.	EGFR/ALK/ROS1/BRAF/KRAS/HER2 Polygene Mutation Testing Kit (Reversible Terminator Sequencing)	National Medical Device Registration Approval No. 20183400408	9/28/2018	Pathology
47	Beijing PINS Medical Co., Ltd.	Implantable Sacral Neuromodulation Pulse Generator Kit	National Medical Device Registration Approval No. 20183120409	9/28/2018	Neurology
48	Beijing PINS Medical Co., Ltd.	Implantable Sacral Neuromodulation Electrode Lead Kit	National Medical Device Registration Approval No. 20183120410	9/28/2018	Neurology
49	Creative Biosciences (Guangzhou) Co., Ltd.	Colosafe® Methylation Detection Kit for Human SDC2 Gene (Real time PCR)	National Medical Device Registration Approval No. 20183400506	11/16/2018	Pathology
50	Amoy Diagnostics Co., Ltd.	AmoyDx® Essential NGS Panel	National Medical Device Registration Approval No. 20183400507	11/16/2018	Pathology
51	Guangdong Zhongneng Accelerator Technology Co., Ltd.	Medical Electronic Linear Accelerator	National Medical Device Registration Approval No. 20183050520	11/28/2018	Radiology
52	Jinshi Biotechnology (Changsu) Co. Ltd.	Valvuloplasty Ring	National Medical Device Registration Approval No. 20183130534	12/7/2018	Cardiovascular
53	Sinovation (Beijing) Medical Technology Co., Ltd.	Neurosurgical Navigation System	National Medical Device Registration Approval No. 20183010598	12/21/2018	Neurology

54	Shanghai United Imaging Healthcare Co. Ltd.	Medical Electronic Linear Accelerator	National Medical Device Registration Approval No. 20183050599	12/24/2018	Radiology
55	Chongqing runze pharmace utical Co. Ltd.	Porous Tantalum Material	National Medical Device Registration Approval No. 20193130001	1/7/2019	Orthopedic
56	Lepu Medical Technology(Beijing)Co.,Lt d.	NeoVas™ Sirolimus-Eluting Bioresorbable Scaffold	National Medical Device Registration Approval No. 20193130093	2/22/2019	Cardiovascular
57	Shenzhen Mindray Bio-Medical Electronics Co., Lt d.	Patient Monitor	National Medical Device Registration Approval No. 20193070154	3/12/2019	General Hospital
58	Shanghai Minimally Invasive Cardiac Medical Technology (Group) Co.,	Abdominal Aortic Stent Graft System	National Medical Device Registration Approval No. 20193130182	3/19/2019	Cardiovascular
59	Beijing Maidi Dingfeng Medical Technology Co., Ltd.	Left Atrial Appendage Closure System	National Medical Device Registration Approval No. 20193130278	4/30/2019	Cardiovascular
60	Shanghai Push Medical Device Co.,Ltd.	Left Atrial Appendage Closure System	National Medical Device Registration Approval No. 20193130279	5/5/2019	Cardiovascular
61	SuperAccuracy (AnHui) Science & Technology Co., Ltd.	Intensity modulated Radiation Therapy Planning System Software	National Medical Device Registration Approval No. 20193210281	5/6/2019	Radiology
62	Shanghai United Imaging Healthcare Co. Ltd.	Digital Mammography System	National Medical Device Registration Approval No. 20193060280	5/6/2019	Radiology
63	Hubei Ruishi Digital Medical Video Technology Co., Ltd.	Positron Emission and X-ray Computed Tomography Scanning System	National Medical Device Registration Approval No. 20193060364	5/31/2019	Radiology
64	Medtronic Inc.	Micra Transcatheter Leadless Pacemaker system	Imported Medical Device Registration Approval No.20193120297	6/11/2019	Cardiovascular

65	MicroPort CardioFlow Medtech Corporation	Transcatheter Aortic Valve System	National Medical Device Registration Approval No. 20193130494	7/10/2019	Cardiovascular
66	Nanjing Wolfman Medical Technology Co., Ltd.	Disposable Intravascular Ultrasound Diagnostic Catheter	National Medical Device Registration Approval No. 20193060601	8/19/2019	Cardiovascular
67	Bobang Fangzhou Medical Technology (Beijing) Co., Ltd.	Non-invasive Blood Glucose Monitoring	National Medical Device Registration Approval No. 20193070602	8/26/2019	General Hospital
68	Chongqing Yongrenxin Medical Instrument Co Ltd	Implantable Left Ventricular Assist System	National Medical Device Registration Approval No. 20193120603	8/26/2019	Cardiovascular
69	Qingdao Zhonghao Biologi cal Engineering Co., Ltd.	Acellular Corneal Graft	National Medical Device Registration Approval No. 20193160679	9/12/2019	Ophthalmic
70	Suzhou RainMed Medical Technology Co., Ltd.	Coronary Angiography-derived Fractional Flow Reserve Measurement System	National Medical Device Registration Approval No. 20193070969	12/9/2019	Cardiovascular
71	Suzhou RainMed Medical Technology Co., Ltd.	Disposable Blood Pressure Transducer	National Medical Device Registration Approval No. 20193070970	12/9/2019	Cardiovascular
72	Shanghai United Imaging Healthcare Co. Ltd.	Positron Emission and X-ray Computed Tomography Scanning System	National Medical Device Registration Approval No. 20193060998	12/17/2019	Radiology
73	Ustar Biotechnologies (Han gzhou) Ltd.	EasyNAT Nucleic Acid Amplification and Detection Analyzer	National Medical Device Registration Approval No. 20193061026	12/23/2019	Molecular Genetics
74	Yida Polestar Medical Tech nology (Suzhou) Co., Ltd.	Puncture Surgery Navigation Device	National Medical Device Registration Approval No. 20203010034	1/14/2020	General & Plastic Surgery
75	Kunlun Medical Cloud Technology	Fractional Coronary Blood Flow Reserve Calculation Software	National Medical Device Registration Approval No. 20203210035	1/14/2020	Radiology

76	Xiamen Feishuo Biotechnology Co., Ltd.	Human EGFR/KRAS/BRAF/PIK3CA/ALK/ROS 1 Gene Mutation Detection Kit	National Medical Device Registration Approval No. 20203400094	1/22/2020	Pathology
77	Suzhou Basecare Medical Device Co. Ltd.	Pre-Implantation Chromosome Aneuploidy Detection Reagent Kit (Semiconductor sequencing method)	National Medical Device Registration Approval No. 20203400181	2/21/2020	Molecular Genetics
78	Shandong Hua'an Biotechnology Co., Ltd.	Sirolimus-Eluting Bioresorbable Scaffold	National Medical Device Registration Approval No. 20203130197	3/4/2020	Cardiovascular
79	Shanghai MicroPort Endovascular MedTech (Group) Co., Ltd.	Drug Coated Balloon Dilatation Catheter	National Medical Device Registration Approval No. 20203130445	4/26/2020	Cardiovascular
80	Shenzhen Vivolight Medical Device & Technology Co.	Intravascular Optical Coherence Tomography Imaging Equipment and accessories	National Medical Device Registration Approval No. 20203060446	4/28/2020	Cardiovascular
81	Boercheng (Beijing) Techn ology Co., Ltd.	RNF180/Septin9 Gene Methylation Detection Kit (PCR fluorescent probe method)	National Medical Device Registration Approval No. 20203400447	4/28/2020	Pathology
82	Hunan Jingyi Medical Tech nology Co., Ltd.	Plasma Surgical Device	National Medical Device Registration Approval No. 20203010474	5/8/2020	General & Plastic Surgery
83	NovoCure Ltd.	Tumour Treating Fields	Imported Medical Device Registration Approval No. 20203090269	5/11/2020	General & Plastic Surgery
84	Edwards Lifesciences LLC	Transcatheter Aortic Valve System	Imported Medical Device Registration Approval No. 20203130291	6/5/2020	Cardiovascular
85	Abbott Vascular	MitraClip System	Imported Medical Device Registration Approval No. 20203130325	6/15/2020	Cardiovascular
86	Shanghai Eagle Eye Medica l Technology Co., Ltd.	Diabetic Retinopathy Eye Bottom Image Auxiliary Diagnostic Software	National Medical Device Registration Approval No. 20203210686	8/7/2020	Ophthalmic

87	Shenzhen Sibionics Technology Co., Ltd.	Diabetic Retinopathy Eye Bottom Image Auxiliary Diagnostic Software	National Medical Device Registration Approval No. 20203210687	8/7/2020	Ophthalmic
88	ZhongAo HuiCheng Technology Co., Ltd.	Hip Joint Coated Ball Head	National Medical Device Registration Approval No. 20203130707	8/20/2020	Orthopedic
89	Ton-Bridge Medical Technology Co., Ltd.	Revascularization Device	National Medical Device Registration Approval No. 20203030728	9/7/2020	Neurology
90	Insight Lifetech Co.,Ltd	VivoCardio® Cardiovascular Pressure Measurement (CPM) System	National Medical Device Registration Approval No. 20203070774	9/29/2020	Cardiovascular
91	Insight Lifetech Co.,Ltd	TRUEPHYSIO® Pressure Microcatheter	National Medical Device Registration Approval No. 20203070775	9/29/2020	Cardiovascular
92	Asclepius Meditec Co., Ltd.	Hydrogen &Oxygen Generator	National Medical Device Registration Approval No. 20203080066	2/2/2020	General Hospital
93	Lanzhou Ximai Memory Alloy Co., Ltd.	Ximai Memory Alloy Nail Foot Fixer	National Medical Device Registration Approval No. 20203130823	10/26/2020	Orthopedic
94	Yukun (Beijing) Network Technology Co., Ltd.	CerebralGo Plus	National Medical Device Registration Approval No. 20203210844	11/3/2020	Radiology
95	Hangzhou Nuohui Health Technology Co. Ltd.	KRAS gene mutation and BMP3/NDRG4 gene methylation and fecalCombination Detection Kit for KRAS Gene Mutation	National Medical Device Registration Approval No. 20203400845	11/9/2020	Pathology
96	Zhejiang Guichuang Medical Technology Co., Ltd.	Paclitaxel-eluting PTA Balloon Dilatation Catheter	National Medical Device Registration Approval No. 20203030857	2020.11.9	Cardiovascular
97	Jiangsu Yitong Biotechnology Co., Ltd.	eton® Peripheral Nerve Graft	National Medical Device Registration Approval No. 20203130898	2020.11.17	Neurology

98	Hangzhou Deepwise & league of PHD Technology Co.,Ltd.	CT Image-aided Detection Software for Intracranial Hemorrhage	National Medical Device Registration Approval No. 20203210920	11/30/2020	Radiology
99	Shanghai Microport Medical (Group) Co., Ltd.	Bridge ® Vertebral Drug-Eluting Stent	National Medical Device Registration Approval No. 20203130971	12/17/2020	Cardiovascular
100	Lifetech Scientific (Shenzhen) Co. Ltd.	Iliac Bifurcation Stent Graft System	National Medical Device Registration Approval No. 20213130022	1/12/2021	Cardiovascular
101	Hunan Apt Medical Device Co., Ltd.	CONQUEROR™ Trap	National Medical Device Registration Approval No. 20213030023	1/13/2021	Cardiovascular
102	AgaMedical Technology Co., Ltd.	Disposable Intravascular Ultrasound Diagnostic Catheter	National Medical Device Registration Approval No. 20213060169	3/9/2021	Cardiovascular
103	Beijing Beifang Tengda Technology Development Co., Ltd.	Single-Use Ureteropelvic Electronic Endoscope	National Medical Device Registration Approval No. 20213060175	3/16/2021	Gastroenterology & Urology
104	Shanghai Outdo Biotech Co. Ltd.	Helicobacter pylori23S rRNA Gene Mutation Detection Kit (RT-PCR)	National Medical Device Registration Approval No. 20213400227	4/1/2021	Microbiology
105	Shenzhen Ruixin Medical Technology Co. Ltd.	Coronary Artery CT Fractional Flow Reserve Calculation Software	National Medical Device Registration Approval No. 20213210270	4/14/2021	Radiology
106	Peijia Medical Limited	Transcatheter Aortic Valve System	National Medical Device Registration Approval No. 20213130275	4/19/2021	Cardiovascular
107	Shenzhen Xianjian Xinkang Medical Electronics Co., Ltd.	Temporary Pacemaker	National Medical Device Registration Approval No. 20213120299	4/26/2021	Cardiovascular
108	Zhejiang Batai Medical Technology Co., Ltd.	Paclitaxel-eluting PTCA Balloon Dilatation Catheter	National Medical Device Registration Approval No. 20213030297	4/30/2021	Cardiovascular

109	Beijing Huifukang Medical Technology Co. Ltd.	Peripheral Nerve Sleeve	National Medical Device Registration Approval No. 20213130298	4/30/2021	Neurology
110	Shanghai MicroPort MedBot (Group) Co., Ltd.	DFVision® 3D Electronic Laparoscope	National Medical Device Registration Approval No. 20213060384	6/4/2021	Gastroenterology & Urology
111	Peijia Medical Limited	Transcatheter Aortic Valve System	National Medical Device Registration Approval No. 20213130464	6/24/2021	Cardiovascular
112	Sequent Medical Inc.	WEB Aneurysm Embolization System	Imported Medical Device Registration Approval No. 20213130233	6/30/2021	Neurology
113	Tianjin Yingtaili Ankang Medical Technology Co., Ltd.	Steep-pulse Therapeutic Instrument	National Medical Device Registration Approval No. 20213090497	7/5/2021	General & Plastic Surgery
114	Beijing Heart Century Medi cal Technology Co., Ltd.	Coronary Artery CT Fractional Flow Reserve Calculation Software	National Medical Device Registration Approval No. 20213210574	7/29/2021	Radiology
115	Sino Medical Sciences Tec hnology Inc.	NOVA DES® Intracranial Drug-Eluting Stent System	National Medical Device Registration Approval No. 20213130575	7/29/2021	Neurology
116	Kossel Medtech Suzhou Co ., Ltd.	Vena Cava Filter	National Medical Device Registration Approval No. 20213130594	8/5/2021	Cardiovascular
117	Beijing Chunlizhengda Medical Instruments Co., Ltd.	Unicompartmental Knee Prosthesis	National Medical Device Registration Approval No. 20213130600	8/6/2021	Orthopedic
118	InnerMedical Co., Ltd.	Endoscopic Ultrasound	National Medical Device Registration Approval No. 20213060608	8/16/2021	Gastroenterology & Urology
119	Shanghai Wallaby Medical Technology Co., Ltd.	Avenir™ Plus Coil System	National Medical Device Registration Approval No. 20213130649	8/18/2021	Neurology

120	MicroPort CardioFlow Medtech Corporation	VitaFlow Liberty™ Transcatheter Aortic Valve and Retrievable Delivery System	National Medical Device Registration Approval No. 20213130655	8/30/2021	Cardiovascular
121	YakeBot (Beijing) Technology Co., Ltd.	Autonomous Dental Implant Robotic System	National Medical Device Registration Approval No. 20213010713	9/13/2021	Dental
122	Huizhou Hydro Caresys Me dical Co., Ltd.	Disposable Debridement Water-powered Knife Head	National Medical Device Registration Approval No. 20213010779	9/27/2021	General & Plastic Surgery
123	Huizhou Hydro Caresys Me dical Co., Ltd.	Hydrodynamic Therapy Equipment	National Medical Device Registration Approval No. 20213010780	9/27/2021	General & Plastic Surgery
124	Suzhou Linatech Medical Science and Technology Co., Ltd.	Medical Electronic Linear Accelerator	National Medical Device Registration Approval No. 20213050789	10/9/2021	Radiology
125	W.L. Gore & Associates, Inc.	GORE®VIABAHN®VBX	Imported Medical Device Registration Approval No. 20213130411	10/19/2021	Cardiovascular
126	Shandong WEGO Surgical Robot Co., Ltd.	Laparoscopic Surgical Robot	National Medical Device Registration Approval No. 20213010848	10/26/2021	Gastroenterology & Urology
127	Beijing Zhongyi Kangwei Medical Instrument Co., Ltd.	Preimplantation Genetic Testing Kit for Aneuploidies (Reverse Termination Sequencing)	National Medical Device Registration Approval No. 20213400868	11/2/2021	Molecular Genetics
128	Shenzhen Sibionics Technology Co., Ltd.	Continuous Glucose Monitoring System	National Medical Device Registration Approval No. 20213070871	11/3/2021	General Hospital
129	Weitai Medical Devices (Hangzhou) Co., Ltd.	Continuous Glucose Monitoring System Equipment	National Medical Device Registration Approval No. 20213070872	11/3/2021	General Hospital
130	Zuoruan Medical Technology (Suzhou) Co., Ltd.	Biodesign Surgisis Hernia Graft	National Medical Device Registration Approval No. 20213130873	11/8/2021	General & Plastic Surgery

131	Suzhou Tongxin Medical Equipment Co., Ltd.	Implantable Left Ventricular Assist System	National Medical Device Registration Approval No. 20213120987	11/24/2021	Cardiovascular
132	Beijing Mihe Medical Devi ces Co., Ltd.	Artificial Cornea	National Medical Device Registration Approval No. 20213161017	12/3/2021	Ophthalmic
133	Shanghai MicroPort Medical (Group) Co., Ltd.	Fontus® Branched Intraoperative Stent System	National Medical Device Registration Approval No. 20213131059	12/14/2021	Cardiovascular
134	MEDTRONIC INC.	Transcatheter Aortic Valve System	Imported Medical Device Registration Approval No. 20213130538	12/24/2021	Cardiovascular
135	Beijing PINS Medical Co., Ltd.	Implantable rechargeable spinal cord stimulator	National Medical Device Registration Approval No. 20223120019	1/10/2022	Neurology
136	Beijing PINS Medical Co., Ltd.	Implantable spinal cord stimulator	National Medical Device Registration Approval No. 20223120020	1/10/2022	Neurology
137	Beijing PINS Medical Co., Ltd.	Implantable spinal cord stimulation electrodes	National Medical Device Registration Approval No. 20223120021	1/10/2022	Neurology
138	Beijing PINS Medical Co., Ltd.	Implantable spinal cord stimulation extension leads	National Medical Device Registration Approval No. 20223120022	1/10/2022	Neurology
139	Beijing PINS Medical Co., Ltd.	Implantable spinal cord stimulation electrodes	National Medical Device Registration Approval No. 20223120023	1/10/2022	Neurology
140	Sinovation (Beijing) Medical Technology Co., Ltd.	Neurosurgical Navigation and Positioning System	National Medical Device Registration Approval No. 20223010024	1/10/2022	Neurology
141	Shanghai MicroPort Medical (Group) Co., Ltd.	Straight Tube Thoracic Aortic Stent Graft System	National Medical Device Registration Approval No. 20223130009	1/6/2022	Cardiovascular

142	Beijing PINS Medical Co., Ltd.	Implantable deep brain stimulation extension lead kit	National Medical Device Registration Approval No. 20223120084	1/20/2022	Neurology
143	Beijing PINS Medical Co., Ltd.	Dual channel rechargeable implantable deep brain stimulation pulse generator kit	National Medical Device Registration Approval No. 20223120085	1/20/2022	Neurology
144	Beijing PINS Medical Co., Ltd.	Implantable deep brain stimulation electrode lead kit	National Medical Device Registration Approval No. 20223120086	1/20/2022	Neurology
145	Beijing PINS Medical Co., Ltd.	Dual channel implantable deep brain stimulation pulse generator kit	National Medical Device Registration Approval No. 20223120087	1/20/2022	Neurology
146	Shanghai MicroPort MedBot (Group) Co., Ltd.	Laparoscopic Surgical System	National Medical Device Registration Approval No. 20223010108	1/25/2022	Gastroenterology & Urology
147	Ankon Medical Technologies (Shanghai) Co., Ltd.	Digestive Tract Vibrating Capsule System	National Medical Device Registration Approval No. 20223090282	2/25/2022	Gastroenterology & Urology
148	Foshan Ruijiatu Medical Technology Co., Ltd.	Portable MagneticResonance Imaging (MRI) of The Brain and Neck	National Medical Device Registration Approval No. 20223060289	3/2/2022	General Hospital
149	Shanghai United Imaging Healthcare Co. Ltd.	CT Image-aided Triage Software for Intracranial Hemorrhage	National Medical Device Registration Approval No. 20223210309	3/9/2022	Radiology
150	XinGaoyi Medical Equipment Co., Ltd.	Magnetic Resonance Imaging System	National Medical Device Registration Approval No. 20223060431	4/2/2022	General Hospital
151	Hangzhou Jianjia Medical Technology Co., Ltd	Joint Replacement Surgical Navigation Positioning System	National Medical Device Registration Approval No. 20223010462	4/6/2022	Orthopedic
152	Suzhou Minimally Invasive Changxing Robot Co., Ltd.	Knee Replacement Surgical Navigation Positioning System	National Medical Device Registration Approval No. 20223010509	4/20/2022	Orthopedic

153	Beijing PINS Medical Co., Ltd.	Spinal cord stimulation test electrodes	National Medical Device Registration Approval No. 20223120511	4/20/2022	Neurology
154	Yuanhua Orthopaedic Robotics Limited	Knee Replacement Surgical Navigation Positioning System	National Medical Device Registration Approval No. 20223010510	4/20/2022	Orthopedic
155	Suzhou Tianhong Shengjie Medical Devices Co., Ltd.	Iliac Venous Stent System	National Medical Device Registration Approval No. 20223130512	4/20/2022	Cardiovascular
156	Medtronic Inc.	TranscatheterLeadless Pacing System	Imported Medical Device Registration Approval No. 20223120231	5/11/2022	Cardiovascular
157	Panorama Hengsheng (Beijing) Science and Technology Co., Ltd.	Intravascular Imaging Device	National Medical Device Registration Approval No. 20223060642	5/18/2022	Cardiovascular
158	Panorama Hengsheng (Beijing) Science and Technology Co., Ltd.	Disposable Intravascular Ultrasound Diagnostic Catheter	National Medical Device Registration Approval No. 20223060641	5/18/2022	Cardiovascular
159	Beijing PINS Medical Co., Ltd.	Patient Programmable Battery Charger	National Medical Device Registration Approval No. 20223120676	5/20/2022	Neurology
160	Hangzhou Weiqiang Medical Technology Co. Ltd.	Thoracic Aortic Stent System	National Medical Device Registration Approval No. 20223130685	5/23/2022	Cardiovascular
161	Beijing Huaco Healthcare Technologies Co., Ltd.	Diagnostic Endoscopic Ultrasound	National Medical Device Registration Approval No. 20223060721	6/2/2022	Gastroenterology & Urology
162	Ningbo Shengjiekang Biotechnology Co., Ltd.	Disposable Cardiac Cryoablation Catheter	National Medical Device Registration Approval No. 20223010763	6/24/2022	General & Plastic Surgery
163	Suzhou Kangduo Robot Co.,Ltd.	Laparoscopic Surgical System	National Medical Device Registration Approval No. 20223010762	6/24/2022	Gastroenterology & Urology

164	Hangzhou Qiming Medical Equipment Co., Ltd.	Transcatheter Pulmonary Valve System	National Medical Device Registration Approval No. 20223130862	7/11/2022	Cardiovascular
165	Aerospace Taixin Technolo gy Co., Ltd.	Implantable Left Ventricular Assist System	National Medical Device Registration Approval No. 20223120892	7/13/2022	Cardiovascular
166	Xian OUR UNITED Corp.	Gamma Knife Stereotactic Radiosurgery System	National Medical Device Registration Approval No. 20223050891	7/13/2022	Radiology
167	LargeV Instrument Corp.,Ltd.	Dual-Energy Cone Beam Computed Tomography—Ultra3D	National Medical Device Registration Approval No. 20223060951	7/19/2022	Ear, Nose, Throat
168	InsightLifetechCo.,Ltd.	Disposable Intravascular Ultrasound Diagnostic Catheter	National Medical Device Registration Approval No. 20223060974	7/25/2022	Cardiovascular
169	InsightLifetechCo.,Ltd.	Intravascular Ultrasound Diagnostic Equipment	National Medical Device Registration Approval No. 20223060975	7/25/2022	Cardiovascular
170	Chengdu Weizhi Medical Equipment Co., Ltd.	Computer Aided Detection Software for Intestinal Polyps in Colonoscopy	National Medical Device Registration Approval No. 20223210981	8/2/2022	Gastroenterology & Urology
171	Ethicon,LLC	SURGICEL Powder and SURGICEL Endoscopic Applicator	Imported Medical Device Registration Approval No. 20223140374	8/3/2022	General & Plastic Surgery
172	BioFire Diagnostics，LLC	FilmArray® Meningitis/Encephalitis (ME) Panel	Imported Medical Device Registration Approval No. 20223400387	8/5/2022	Microbiology
173	Beijing Biosis Healing Biological Technology Co., Ltd.	AnastomosisReinforcement Patch	National Medical Device Registration Approval No. 20223130983	8/3/2022	General & Plastic Surgery
174	Hangzhou Yahui Biotechnology Co., Ltd.	Medical adhesives	National Medical Device Registration Approval No. 20223021122	8/29/2022	General & Plastic Surgery

175	Tencent Healthcare (Shenzhen) Co., Ltd.	Fundus Image-aided Detection Software For Chronic Glaucoma-like Optic Neuropathy	National Medical Device Registration Approval No. 20223211140	8/31/2022	Ophthalmic
176	Shanghai United Imaging Healthcare Co. Ltd.	Magnetic Resonance Imaging System	National Medical Device Registration Approval No. 20223061141	8/31/2022	General Hospital
177	Shandong Jiwei Medical Pr oducts Co., Ltd.	Umelimus Coated Balloon Catheter	National Medical Device Registration Approval No. 20223031247	9/19/2022	Cardiovascular
178	Shanghai APACTRON Particle Equipment Co., Ltd.	Proton Therapy System	National Medical Device Registration Approval No. 20223051290	9/26/2022	Radiology
179	Dongguan Coldwell Medical Instrument co., LTD.	MicroPort® Kewei Membrane Oxygenator	National Medical Device Registration Approval No. 20223101297	9/27/2022	General Hospital
180	Qianglian Zhichuang (Beijing) Technology Co., Ltd.	UKnow® Aneurysm Surgery Planning Software	National Medical Device Registration Approval No. 20223211346	10/11/2022	Neurology
181	Aike Medical Devices (Beijing) Co., Ltd.	Flex Embolization Device	National Medical Device Registration Approval No. 20223131392	10/24/2022	Neurology
182	Aibo Nord (Beijing) Medical Technology Co., Ltd.	Multifocal Aspheric Diffractive Intraocular Lens	National Medical Device Registration Approval No. 20223161440	10/28/2022	Ophthalmic
183	Hangzhou Deno Electrophysiology Medical Technology Inc.	Left Atrial Appendage Closure System	National Medical Device Registration Approval No. 20223131498	11/9/2022	Cardiovascular
184	Jiangsu Baiyouda Life Technology Co.,Ltd.	Artificial Blood Vessels	National Medical Device Registration Approval No. 20223131515	11/17/2022	Cardiovascular
185	Shanghai MicroPort EP MedTech Co.,Ltd.	Single-use pressure monitoring magnetic positioning radiofrequency	National Medical Device Registration Approval No. 20223011571	12/1/2022	Cardiovascular

186	Shenzhen Jingfeng MEDICAL Technology Co., Ltd.	Laparoscopic Surgical System	National Medical Device Registration Approval No. 20223011623	12/14/2022	Gastroenterology & Urology
187	SonoScape Medical Corp.	Intravascular Ultrasound Diagnostic Equipment	National Medical Device Registration Approval No. 20223061658	12/14/2022	Cardiovascular
188	Shanghai Aisheng Biology Medical Treatment Technology Co.	Disposable Intravascular Ultrasound Diagnostic Catheter	National Medical Device Registration Approval No. 20223061659	12/14/2022	Cardiovascular
189	Shanghai United Imaging Healthcare Co. Ltd.	Medical Angiography X-ray Machine	National Medical Device Registration Approval No. 20223061821	12/30/2022	Radiology
190	Shenzhen Comen Medical Instruments Co., Ltd.	Patient monitor	National Medical Device Registration Approval No. 20233070003	1/5/2023	General Hospital
191	Medtronic MiniMed	Hybrid Closed Loop Insulin Delivery System (MiniMed 670G BLE)	Imported Medical Device Registration Approval No. 20233140061	2/27/2023	Clinical Chemistry
192	Beijing IfmSoft Co., Ltd	Hemodialysis urea clearance calculation software	National Medical Device Registration Approval No. 20233210219	2/27/2023	Gastroenterology & Urology
193	Ubiosis Co., Ltd.	Collagen Scaffold for Cartilage Repair (COLTRIX CartiRegen)	Imported Medical Device Registration Approval No. 20233130129	4/4/2023	Orthopedic
194	Sinovation (Beijing) Medical Technology Co., Ltd.	Magnetic Resonance Monitoring Semiconductor Laser Treatment Equipment	National Medical Device Registration Approval No. 20233010449	4/4/2023	Neurology
195	Shanghai Pulse Medical Technology Co., Ltd.	Coronary Artery CT Fractional Flow Reserve Calculation Software	National Medical Device Registration Approval No. 20233210450	4/4/2023	Radiology
196	Sinovation (Beijing) Medical Technology Co., Ltd.	Disposable Laser Optical Fiber Kit	National Medical Device Registration Approval No. 20233010485	4/13/2023	Neurology

197	Alcon Laboratories, Inc.	Artificial Lens	Imported Medical Device Registration Approval No. 20233160146	4/17/2023	Ophthalmic
198	Suzhou RainMed Medical Technology Co., Ltd.	Coronary Function Measurement System	National Medical Device Registration Approval No. 20233070520	4/20/2023	Cardiovascular
199	Beijing AK Medical Co., Ltd.	Metal Additive Manufacturing Thoracolumbar Fusion Matching Prosthesis System	National Medical Device Registration Approval No. 20233130524	4/21/2023	Orthopedic
200	Rongsheng (Nanjing) Medical Technology Co., Ltd.	Self-expanding Radioactive Seed-Carried Biliary Stent	National Medical Device Registration Approval No. 20233130621	5/11/2023	General & Plastic Surgery
201	Wuhan ENDOANGEL Medical Technology Co., Ltd.	Computer Aided Detection Software for Intestinal Polyps in Lower Gastrointestinal Endoscopy	National Medical Device Registration Approval No. 20233210629	5/12/2023	Gastroenterology & Urology
202	Conavi Medical Inc.	Intravascular Imaging Device	Imported Medical Device Registration Approval No. 20233060200	5/16/2023	Cardiovascular
203	Shanghai United Imaging Healthcare Co. Ltd.	Radiotherapy Planning Software	National Medical Device Registration Approval No. 20233210665	5/23/2023	Radiology
204	Tencent Healthcare (Shenzhen) Co., Ltd.	Computer-aided Detection Software for Electronic Endoscope for Colon Polyps	National Medical Device Registration Approval No. 20233210707	6/1/2023	Gastroenterology & Urology
205	Corindus Inc.	Coronary Interventional Surgical Control System with One-off Accessories	Imported Medical Device Registration Approval No. 20233010226	6/1/2023	Cardiovascular
206	Corindus Inc.	Coronary Interventional Surgical Control System	Imported Medical Device Registration Approval No. 20233010225	6/1/2023	Cardiovascular
207	Lanzhou Ion Therapy Co., Ltd.	Carbon Ion Therapy System	National Medical Device Registration Approval No. 20233050708	6/2/2023	Radiology

208	Shenzhen Core Medical Technology Co., Ltd.	Implantable Left Ventricular Assist System	National Medical Device Registration Approval No. 20233120716	6/5/2023	Cardiovascular
209	Shanghai Magic Medical Technology Co., Ltd.	Multimodal Tumor Treatment System	National Medical Device Registration Approval No. 20233010773	6/8/2023	General & Plastic Surgery
210	True Health (Beijing) Medical Technology Co., Ltd.	Implantable Sacral Nerve Stimulator	National Medical Device Registration Approval No. 20233120807	6/14/2023	Neurology
211	True Health (Beijing) Medical Technology Co., Ltd.	Implantable Sacral Nerve Stimulation Extension Wire	National Medical Device Registration Approval No. 20233120808	6/14/2023	Neurology
212	True Health (Beijing) Medical Technology Co., Ltd.	Implantable Sacral Nerve Stimulation Electrode	National Medical Device Registration Approval No. 20233120809	6/14/2023	Neurology
213	True Health (Beijing) Medical Technology Co., Ltd.	Puncture Surgical Navigation Positioning System	National Medical Device Registration Approval No. 20233010810	6/15/2023	General & Plastic Surgery
214	Suzhou MicroPort Orthopedics Medical Technology Co., Ltd.	Zirconium Niobium Alloy Femoral Head	National Medical Device Registration Approval No. 20233130813	6/15/2023	Orthopedic
215	Shenzhen Huikang Precisio n Instrument Co.,Ltd.	Shockwave Therapy Machine	National Medical Device Registration Approval No. 20233090834	6/20/2023	Gastroenterology & Urology
216	Beijing Surgerii Robotics Company	Single-port Laparoscope Surgery System	National Medical Device Registration Approval No. 20233010833	6/20/2023	Gastroenterology & Urology
217	Advanced Surgical Retractor Systems, Inc.	Head and Neck X-ray Stereotactic Radiosurgery System	Imported Medical Device Registration Approval No. 20233050263	6/20/2023	Radiology
218	Beijing Tinavi Medical Technologies Co., Ltd.	Knee Replacement Surgical Navigation Positioning System	National Medical Device Registration Approval No. 20233010962	7/13/2023	Orthopedic

219	Alcon Laboratories, Incorporated	Intraocular lenses	Imported Medical Device Registration Approval No. 20233160317	7/20/2023	Ophthalmic
220	Suzhou Innomed Medical Device Co., Ltd.	Venous Stent System	National Medical Device Registration Approval No. 20233131034	7/28/2023	Cardiovascular
221	Xian OUR UNITED Corp.	Medical Electronic Linear Accelerator	National Medical Device Registration Approval No. 20233051067	8/1/2023	Radiology
222	Conavi Medical Inc.	Disposable Intravascular Ultrasound Diagnostic Catheter	Imported Medical Device Registration Approval No. 20233060333	8/1/2023	Cardiovascular
223	Shanghai Aikangte Medical Technology Co., Ltd.	Rigid Scleral Contact Lens	National Medical Device Registration Approval No. 20233161081	8/3/2023	Ophthalmic
224	Hangzhou Lancet Robotics company	Hip Replacement Navigation Positioning System	National Medical Device Registration Approval No. 20233011155	8/10/2023	Orthopedic
225	Wuhan Verlmagin Medical Technology Co., Ltd	Magnetic Resonance Imaging System	National Medical Device Registration Approval No. 20233061160	8/16/2023	General Hospital
226	Rapid Medical Ltd.	Tigertriever® Revascularization Device	Imported Medical Device Registration Approval No. 20233030369	8/17/2023	Neurology
227	Shanghai MicroPort EP MedTech Co.,Ltd.	Balloon Cryoablation Catheter	National Medical Device Registration Approval No. 20233011225	8/23/2023	Cardiovascular
228	Shanghai MicroPort EP MedTech Co.,Ltd.	Cryoablation Equipment	National Medical Device Registration Approval No. 20233011226	8/23/2023	Cardiovascular
229	Shanxi Jinbo Bio-pharmaceutical Co., Ltd.	Recombinant Humanized Type III Collagen Solution	National Medical Device Registration Approval No. 20233131245	8/25/2023	General & Plastic Surgery

230	Shanghai Hanyu Medical Technology Co., Ltd.	Mitral Valve Clamp System	National Medical Device Registration Approval No. 20233131292	9/7/2023	Cardiovascular
231	Nanjing Shihe Medical Equipment Co., Ltd.	Non-small Cell Lung Cancer Tumour Mutational Burden Test (NSCLC TMB) Kit (Reverse Termination Sequencing)	National Medical Device Registration Approval No. 20233401452	10/12/2023	Pathology
232	Siemens Healthcare GmbH	X-Ray Computed Tomography	Imported Medical Device Registration Approval No. 20233060455	10/16/2023	Radiology
233	Beijing Changmugu Medic al Technology	Joint Replacement Surgery Simulation Software	National Medical Device Registration Approval No. 20233211543	10/23/2023	Orthopedic
234	Varian Medical Systems Inc.	Proton Therapy System	Imported Medical Device Registration Approval No. 20233050480	11/1/2023	Radiology
235	Beijing Novel Medical Equipment Ltd.	Single Photon Emission and X-ray Computed Tomography Imaging System	National Medical Device Registration Approval No. 20233061628	11/7/2023	Radiology
236	Kontour(Xi'an) Medical Technology Co., Ltd.	Additive Manufacturing Polyetheretherketone Skull Defect Repair Prosthesis	National Medical Device Registration Approval No. 20233131652	11/9/2023	Orthopedic
237	Naton Biotechnology (Beijing) Co., Ltd.	Additive Manufacturing Matching Knee Prosthesis	National Medical Device Registration Approval No. 20233131698	11/17/2023	Orthopedic
238	Shenzhen Jingfeng Medical Technology Co., Ltd.	Laparoscopic single-port surgical system	National Medical Device Registration Approval No. 20233011753	11/24/2023	Gastroenterology & Urology
239	Jiangsu Tingsheng Technology Co., Ltd.	Disposable Intracardiac Ultrasound Imaging Catheter	National Medical Device Registration Approval No. 20233061761	11/28/2023	Cardiovascular
240	Neo Modulus (Suzhou) Medical Sci-Tech Co., Ltd.	Gelatin Polycaprolactone Layered Gingival Repair Membrane	National Medical Device Registration Approval No. 20233171776	11/29/2023	Dental

241	Hangzhou Valgen MedTech Co., Ltd.	Transcatheter Mitral Valve Repair System	National Medical Device Registration Approval No. 20233131775	11/29/2023	Cardiovascular
242	Cryofocus Medtech (Shanghai) Co., Ltd.	Cryoablation Equipment	National Medical Device Registration Approval No. 20233011815	12/4/2023	Cardiovascular
243	Cryofocus Medtech (Shanghai) Co., Ltd.	Balloon Cryoablation Catheter	National Medical Device Registration Approval No. 20233011816	12/4/2023	Cardiovascular
244	Beijing Rossum Robot Technology Co., Ltd.	Robot-Assisted Fracture Reduction System for Pelvic Fracture	National Medical Device Registration Approval No. 20233011923	12/8/2023	Orthopedic
245	Suzhou Origin Medical Technology Co., Ltd.	Degradable Magnesium Closing Clip	National Medical Device Registration Approval No. 20233021931	12/13/2023	General & Plastic Surgery
246	BioNTech Diagnostics GmbH	MammaTyper® Kit (RT-PCR)	Imported Medical Device Registration Approval No. 20233400600	12/21/2023	Pathology
247	Wuxi Pamu Medical Technology Co., Ltd.	Disposable Annular Pulmonary Artery Denervation Catheter	National Medical Device Registration Approval No. 20233012011	12/22/2023	Cardiovascular
248	Sichuan Jinjiang Electronic Medical Device Tech Co., Ltd.	Pulsed Field Ablation Catheter	National Medical Device Registration Approval No. 20233012053	12/26/2023	Cardiovascular
249	Sichuan Jinjiang Electronic Medical Device Tech Co., Ltd.	Pulsed Field Ablation Generator	National Medical Device Registration Approval No. 20233012051	12/26/2023	Cardiovascular
250	Varian Medical Systems Inc.	Proton Therapy System	Imported Medical Device Registration Approval No. 20233050613	12/26/2023	Radiology
251	Hangzhou Tongee Medical Technology Co., Ltd.	Gastric Bypass Stent System	National Medical Device Registration Approval No. 20243130104	1/17/2024	Gastroenterology & Urology

252	ShenZhen Sciarray Biotechnology Co., Ltd.	IFI44L Gene Methylation Detection Kit (PCR-Melting Curve Method)	National Medical Device Registration Approval No. 20243400284	2/5/2024	Pathology
253	Hangzhou Deepwise	CT Image-aided Detection Software for Intracranial Aneurysms	National Medical Device Registration Approval No. 20243210396	2/29/2024	Radiology
254	IntuitiveSurgical, Inc.	Bronchial Navigation System Supporting Passive Accessories	Imported Medical Device Registration Approval No. 20242080127	3/8/2024	General & Plastic Surgery
255	Hangzhou Deno Electrophysiology Medical Technology Inc.	Disposable Cardiac Pulsed Electric Field Ablation Catheter	National Medical Device Registration Approval No. 20243010461	3/8/2024	Cardiovascular
256	IntuitiveSurgical, Inc.	Bronchoscope Navigation Optical Fiber Positioning Catheter Guide	Imported Medical Device Registration Approval No. 20242010154	3/20/2024	General & Plastic Surgery
257	IntuitiveSurgical, Inc.	Bronchoscope Navigation Optical Fiber Positioning Catheter	Imported Medical Device Registration Approval No. 20243010156	3/20/2024	General & Plastic Surgery
258	IntuitiveSurgical, Inc.	Bronchoscope Navigation Visualization Probe	Imported Medical Device Registration Approval No. 20242060153	3/20/2024	General & Plastic Surgery
259	IntuitiveSurgical, Inc.	Bronchoscope Navigation Operation Control System	Imported Medical Device Registration Approval No. 20243010155	3/20/2024	General & Plastic Surgery
260	Hangzhou Jingce Medical Technology Co., Ltd	Combined Glucometer	National Medical Device Registration Approval No. 20243070662	4/11/2024	General Hospital
261	Jiangsu SysDiagno Biotech Co., Ltd.	Oligosaccharide Chain Detection Kit (Fluorophore-Assisted Carbohydrate Electrophoresis)	National Medical Device Registration Approval No. 20243400663	4/11/2024	Pathology
262	magAssist Inc. (Suzhou)	MoyoAssist® Extracorporeal Ventricular Assist Device	National Medical Device Registration Approval No. 20243100773	4/26/2024	Cardiovascular

263	magAssist Inc. (Suzhou)	MoyoAssist® Extracorporeal Ventricular Assist Pump Head and Circuit	National Medical Device Registration Approval No. 20243100774	4/26/2024	Cardiovascular
264	Medtronic, Inc.	Symplicity Spyral™ Multi-Electrode Renal Denervation Catheter	Imported Medical Device Registration Approval No. 20243010242	4/30/2024	Cardiovascular
265	Medtronic, Inc.	Symplicity G3™ Renal Denervation RF Generator	Imported Medical Device Registration Approval No. 20243010243	4/30/2024	Cardiovascular
266	Beijing PINS Medical Co., Ltd.	Dual Channel Rechargeable Implantable Deep Brain Stimulation Pulse Generator Kit	National Medical Device Registration Approval No. 20243120979	5/23/2024	Neurology
267	Beijing PINS Medical Co., Ltd.	Dual Channel Implantable Deep Brain Stimulation Pulse Generator Kit	National Medical Device Registration Approval No. 20243120980	5/23/2024	Neurology
268	Beijing PINS Medical Co., Ltd.	Implantable Deep Brain Stimulation Electrode Lead Kit	National Medical Device Registration Approval No. 20243120981	5/23/2024	Neurology
269	Beijing PINS Medical Co., Ltd.	Implantable Deep Brain Stimulation Extension Lead Kit	National Medical Device Registration Approval No. 20243120982	5/23/2024	Neurology
270	Shanghai Tengfu Medical Technology Co., Ltd	Pulmonary Artery Thrombectomy Stent System	National Medical Device Registration Approval No. 20243031010	5/31/2024	Cardiovascular
271	Beijing Ailin Medical Technology Co., Ltd.	Transjugular Intrahepatic Puncture Instrument	National Medical Device Registration Approval No. 20243031052	5/31/2024	Cardiovascular
272	Dinova Medtech (Hong Kong) Co., Limited	Portable Radiofrequency Needle For Transseptal Puncture	National Medical Device Registration Approval No. 20243011095	6/7/2024	Cardiovascular
273	Beijing Zhiyuan Huitu Technology Co., Ltd	Fundus Image-aided Detection Software For Fundus Lesions	National Medical Device Registration Approval No. 20243211109	6/18/2024	Ophthalmic

274	IntuitiveSurgical, Inc.	Needles of Bronchoscope Navigation Operation Control System	National Medical Device Registration Approval No. 20242020316	6/19/2024	General & Plastic Surgery
275	Shanghai Antike Medical Technology Co., Ltd.	Cryoablation System	National Medical Device Registration Approval No. 20243011210	7/4/2024	Cardiovascular
276	FARAPULSE, Inc.	FARAPULSE™ Pulsed Field Ablation Catheter	Imported Medical Device Registration Approval No. 20243010355	7/4/2024	Cardiovascular
277	FARAPULSE, Inc.	FARAPULSE™ Pulsed Field Ablation System	Imported Medical Device Registration Approval No. 20243090354	7/4/2024	Cardiovascular
278	Hangzhou Weiqiang Medical Technology Co. Ltd.	Iliac Venous Stent System	National Medical Device Registration Approval No. 20243131244	7/11/2024	Cardiovascular
279	Sonosemi Medical Co., Ltd.	Disposable Intracardiac Ultrasound Imaging Catheter	National Medical Device Registration Approval No. 20243061274	7/17/2024	Cardiovascular
280	Sonosemi Medical Co., Ltd.	Portable Ultrasound Machine	National Medical Device Registration Approval No. 20243061271	7/17/2024	Cardiovascular
281	FARAPULSE, Inc.	Disposable Adjustable Elbow Catheter Sheath	Imported Medical Device Registration Approval No. 20243030396	7/24/2024	Cardiovascular
282	MicroPort Scientific Corpor ation	Firesorb® Sirolimus-Eluting Bioresorbable Scaffold	National Medical Device Registration Approval No. 20243131356	7/30/2024	Cardiovascular
283	Suzhou Xinmai Medical Instrument Co., Ltd	Renal Denervation Generator	National Medical Device Registration Approval No. 20243011383	8/5/2024	Cardiovascular
284	Suzhou Xinmai Medical Instrument Co., Ltd	Disposable Renal Denervation Catheter	National Medical Device Registration Approval No. 20243011384	8/5/2024	Cardiovascular

285	Shanghai Golden Leaf Med Tec Co., Ltd.	Renal Denervation Generator	National Medical Device Registration Approval No. 20243011387	8/5/2024	Cardiovascular
286	Shanghai Golden Leaf Med Tec Co., Ltd.	Disposable Reticular Renal Denervation Catheter	National Medical Device Registration Approval No. 20243011385	8/5/2024	Cardiovascular
287	Shanghai Antike Medical Technology Co., Ltd.	Balloon Cryoablation Catheter	National Medical Device Registration Approval No. 20243011414	8/5/2024	Cardiovascular
288	Shanghai Laiye Medical Technology Co., Ltd.	Transcatheter Aortic Valve System	National Medical Device Registration Approval No. 20243131459	8/14/2024	Cardiovascular
289	Shanghai Bluevascular MedTech Co., Ltd.	Venous Stent System	National Medical Device Registration Approval No. 20243131528	8/22/2024	Cardiovascular
290	Beijing Balance Medical Technology Co., Ltd.	Transcatheter Aortic Valve System	National Medical Device Registration Approval No. 20243131529	8/22/2024	Cardiovascular
291	Boston Scientific Corporation	Jetstream™ Atherectomy Control System	Imported Medical Device Registration Approval No. 20243010448	8/22/2024	Cardiovascular
292	Boston Scientific Corporation	Jetstream™ Atherectomy Catheter For Peripheral Vascular	Imported Medical Device Registration Approval No. 20243010449	8/22/2024	Cardiovascular
293	Fujirebio, Inc.	Hepatitis B Surface Antigen (HBsAg) Detection Kit (Chemiluminescence Immunoassay Method)	Imported Medical Device Registration Approval No. 20243400480	8/30/2024	Microbiology
294	TrueHealth (Zhuhai) Medical Technology Co., Ltd.	Navigation and Positioning Microwave Ablation System	National Medical Device Registration Approval No. 20243011651	8/30/2024	General & Plastic Surgery
295	Medtronic, Inc.	PulseSelect™ Pulsed Field Ablation Generator	Imported Medical Device Registration Approval No. 20243010482	9/5/2024	Cardiovascular

296	Medtronic, Inc.	PulseSelect™ Pulsed Field Ablation Catheter	Imported Medical Device Registration Approval No. 20243010487	9/10/2024	Cardiovascular
297	Shanghai United Imaging Healthcare Co. Ltd.	CT Angiogram-aided Detection Software for Intracranial Aneurysms	National Medical Device Registration Approval No. 20243211923	9/25/2024	Radiology
298	Lanzhou Ion Therapy Co., Ltd.	Carbon Ion Therapy System	National Medical Device Registration Approval No. 20243051929	9/29/2024	Radiology
299	YBNX Medical Co., Ltd.	Knee Prosthesis System	National Medical Device Registration Approval No. 2024313215	10/31/2024	Orthopedic
300	Shanghai HeartCare Medical Technology Corporation Limited	Intracranial Stent for Aneurysm Embolization	National Medical Device Registration Approval No. 20243132152	10/31/2024	Neurology
301	Sinovation (Beijing) Medical Technology Co., Ltd.	Neurosurgical Planning Software	National Medical Device Registration Approval No. 20243212209	11/6/2024	Neurology
302	Boston Scientific Neuromodulation Corporation	DBS Directional Lead	Imported Medical Device Registration Approval No. 20243120580	11/6/2024	Neurology
303	Beijing Ansong Technology Co., Ltd.	Zirconia Ceramic Femoral Head	National Medical Device Registration Approval No. 20243132300	11/21/2024	Orthopedic
304	MicroPort Soaring CRM (Shanghai) Co., Ltd.	Implantable Cardiac Pacing Lead	National Medical Device Registration Approval No. 20243122320	11/21/2024	Cardiovascular
305	Shenzhen Mindray Bio-Medical Electronics Co., Lt d.	Ultrasound Diagnostic System	National Medical Device Registration Approval No. 20243062383	11/27/2024	General Hospital
306	Hangzhou Ruidi Biotechnology Co., Ltd.	Disposable high-pulse electrode ablation needle	National Medical Device Registration Approval No. 20243092384	11/27/2024	General & Plastic Surgery

307	Hangzhou Ruidi Biotechnology Co., Ltd.	High-Pulse Electric Field Ablation System	National Medical Device Registration Approval No. 20243092385	11/27/2024	General & Plastic Surgery
308	Varian Medical Systems Inc.	Proton Therapy System	Imported Medical Device Registration Approval No. 20243050647	12/5/2024	Radiology
309	Mevion Medical Systems, Inc.	Proton Therapy System	Imported Medical Device Registration Approval No. 20243050656	12/13/2024	Radiology
310	Shanghai Hongtong Industrial Co., Ltd.	Cardiac Pulsed Electric Field Ablation Instrument	National Medical Device Registration Approval No. 20243012477	12/13/2024	Cardiovascular
311	Shanghai Changdi Medical Technology Co., Ltd.	Artificial Blood Vessel	National Medical Device Registration Approval No. 20243132518	12/16/2024	Cardiovascular
312	Lifetech Scientific (Shenzhen) Co. Ltd.	Aortic Covered Stent Fenestration System	National Medical Device Registration Approval No. 20243032519	12/16/2024	Cardiovascular
313	Changsha Huiwei Intelligent Medical Technology Co., Ltd.	Intestinal Polyp Electronic Lower Gastrointestinal Endoscopy Image-Assisted Detection Software	National Medical Device Registration Approval No. 20243212606	12/17/2024	Gastroenterology & Urology
314	Shanghai MicroPort Melody Medical Technology Co., Ltd.	Rotational Atherectomy Intervention Therapy Instrument	National Medical Device Registration Approval No. 20243012607	12/17/2024	Cardiovascular
315	Shanghai MicroPort Melody Medical Technology Co., Ltd.	Disposable Coronary Rotational Atherectomy Catheter	National Medical Device Registration Approval No. 20243012608	12/17/2024	Cardiovascular
316	Hunan Apt Medical Device Co., Ltd.	Disposable Magnetoelectric Positioning Pressure Monitoring Pulsed Electric Field Ablation Catheter	National Medical Device Registration Approval No. 20243012609	12/17/2024	Cardiovascular
317	Aibo Nord (Beijing) Medical Technology Co., Ltd.	Phakic Intraocular Lens	National Medical Device Registration Approval No. 20253160001	1/6/2025	Ophthalmic

318	Synaptic MEDICAL TECHNOLOGIES(Beijing) Co., Ltd.	Cryoablation Instrument	National Medical Device Registration Approval No. 20253010184	1/20/2025	Cardiovascular
319	Wuhan Synaptic Biotechnology Co., Ltd.	Human CDO1/AJAP1/GALR1 Gene Methylation Detection Kit (Real time PCR)	National Medical Device Registration Approval No. 20253400185	1/20/2025	Pathology
320	Tianjin Century Healthcare Biomedical Engineering Co., Ltd.	Multifocal Intraocular Lens	National Medical Device Registration Approval No. 20253160240	1/24/2025	Ophthalmic
321	TriReme Medical,LLC	Paclitaxel-Coated Peripheral Balloon Catheter	Imported Medical Device Registration Approval No. 20253030062	1/24/2025	Cardiovascular
322	Promega (Shanghai) Bioproducts Co., Ltd.	Microsatellite Instability (MSI) Detection Kit (Real time PCR-Capillary Electrophoresis Method)	National Medical Device Registration Approval No. 20253400266	1/24/2025	Pathology
323	Biosense Webster (Israel) Ltd.	TRUPULSE Generator	Imported Medical Device Registration Approval No. 20253010069	1/26/2025	Cardiovascular
324	Biosense Webster (Israel) Ltd.	Bi-directional Catheter	Imported Medical Device Registration Approval No. 20253010070	1/26/2025	Cardiovascular
325	Hangzhou Duanyou Medical Technology Co., Ltd.	Transcatheter Mitral Valve Repair System	National Medical Device Registration Approval No. 20253130304	2/8/2025	Cardiovascular
326	Lifetech Scientific (Shenzhen) Co. Ltd.	Aortic Stent Graft System	National Medical Device Registration Approval No. 20253130305	2/8/2025	Cardiovascular
327	Synaptic MEDICAL TECHNOLOGIES(Beijing) Co., Ltd.	Disposable Balloon-type Cryoablation Catheter	National Medical Device Registration Approval No. 20253010391	2/18/2025	Cardiovascular
328	MediBeacon Inc.	Percutaneous Glomerular Filtration Rate (GFR) Measurement Device	Imported Medical Device Registration Approval No. 20253070094	2/18/2025	Gastroenterology & Urology

329	Shanghai Xuanyu Medical Device Co., Ltd.	Cardiac Pulsed Electric Field Ablation Instrument	National Medical Device Registration Approval No. 20253010430	2/26/2025	Cardiovascular
330	Shanghai Xuanyu Medical Device Co., Ltd.	Disposable Cardiac Pulsed Electric Field Ablation Catheter	National Medical Device Registration Approval No. 20253010431	2/26/2025	Cardiovascular
331	Shanghai Huihe Medical Technology Co., Ltd.	Transcatheter Tricuspid Annuloplasty System	National Medical Device Registration Approval No. 20253130510	3/7/2025	Cardiovascular
332	Shenzhen Robo Medical Technology Co., Ltd.	Endoscopic Surgical Instrument Control Device	National Medical Device Registration Approval No. 20253010539	3/11/2025	Gastroenterology & Urology
333	Inari Medical，Inc	ClotTriever Thrombectomy System	Imported Medical Device Registration Approval No. 20253030131	3/14/2025	Cardiovascular
334	Beijing Weimai Medical Equipment Co., Ltd.	Coronary Interventional Surgery Control System	National Medical Device Registration Approval No. 20253010587	3/21/2025	Cardiovascular
335	Hunan Apt Medical Device Co., Ltd.	Disposable Pressure Monitoring Radiofrequency Ablation Catheter	National Medical Device Registration Approval No. 20253010659	3/26/2025	Cardiovascular
336	Shanghai Shangyang Medical Technology Co., Ltd.	Cardiac Pulsed Electric Field Ablation Equipment	National Medical Device Registration Approval No. 20253010702	4/1/2025	Cardiovascular
337	Shanghai Shangyang Medical Technology Co., Ltd.	Disposable Magnetoelectric Positioning Cardiac Pulsed Electric Field Ablation Catheter	National Medical Device Registration Approval No. 20253010703	4/1/2025	Cardiovascular
338	Tianjin Yingtaili Ankang Medical Technology Co., Ltd.	Cardiac Pulsed Electric Field Ablation Instrument	National Medical Device Registration Approval No. 20253010705	4/1/2025	Cardiovascular
339	Tianjin Yingtaili Ankang Medical Technology Co., Ltd.	Disposable Cardiac Pulsed Electric Field Ablation Catheter	National Medical Device Registration Approval No. 20253010704	4/1/2025	Cardiovascular

340	Jinshi Biotechnology (Changsu) Co. Ltd.	Transcatheter Aortic Valve System	National Medical Device Registration Approval No. 20253130729	4/9/2025	Cardiovascular
341	Beijing Percutek Therapeutics Inc.	Integrated Intraoperative Vascular Graft Stent System	National Medical Device Registration Approval No. 20253130730	4/9/2025	Cardiovascular
342	BREATH MEDICAL Co. Ltd.	Degradable Ear and Nose Hemostatic Cotton	National Medical Device Registration Approval No. 20253140731	4/9/2025	Ear, Nose, Throat
343	Jiangsu Nuanyang Medical Equipment Co., Ltd.	Flex Embolization Device	National Medical Device Registration Approval No. 20253130732	4/9/2025	Neurology
344	Beijing WANJIE Medical Device Corporation Limited	Artificial Ligament	National Medical Device Registration Approval No. 20253130733	4/9/2025	General & Plastic Surgery
345	Medtronic Inc.	Extravascular Implantable Cardiac Defibrillation Electrode Lead	Imported Medical Device Registration Approval No. 20253120181	4/17/2025	Cardiovascular
346	Medtronic Inc.	Extravascular Implantable Cardiac Defibrillation Electrode Lead Introducer	Imported Medical Device Registration Approval No. 20253120182	4/17/2025	Cardiovascular
347	Sichuan Jinjiang Electronic Medical Device Technology Co., Ltd.	PulsedFA® FocalPoint Disposable Magnetic Localization Pressure Monitoring Pulse Electric Field Ablation	National Medical Device Registration Approval No. 20253010788	4/21/2025	Cardiovascular
348	Suzhou Aikemai Medical Technology Co., Ltd.	AccuBlator® Multi-channel Pulse Electric Field Ablation Instrument	National Medical Device Registration Approval No. 20253010860	4/29/2025	Cardiovascular
349	Suzhou Aikemai Medical Technology Co., Ltd.	AccuPulse® Disposable Cardiac Pulse Electric Field Ablation Catheter	National Medical Device Registration Approval No. 20253010879	4/29/2025	Cardiovascular
350	Suzhou 3N Biological Technology Co., Ltd.	Rigid Contact Lens Care Device	National Medical Device Registration Approval No. 20253160887	4/29/2025	Ophthalmic

351	Beijing Balance Medical Technology Co., Ltd.	RENATO® Transcatheter Valve-in-Valve System	National Medical Device Registration Approval No. 20253130951	5/14/2025	Cardiovascular
352	Shenzhen Zhongke Jingcheng Medical Technology Co., Ltd.	Magnesium-containing Degradable Polymer Bone Repair Material	National Medical Device Registration Approval No. 20253130952	5/14/2025	Orthopedic
353	Lifetech Scientific (Shenzhen) Co. Ltd.	Aortic Arch Stent Graft System	National Medical Device Registration Approval No. 20253130950	5/14/2025	Cardiovascular
354	Koka (Nantong) Lifesciences Co., Ltd.	Transcatheter Mitral Valve Repair System	National Medical Device Registration Approval No. 20253130967	5/27/2025	Cardiovascular
355	Medtronic Inc.	Extravascular Implantable Cardioverter Defibrillator	Imported Medical Device Registration Approval No. 20253120253	6/6/2025	Cardiovascular
356	Shenzhen Robo Medical Technology Co., Ltd.	Disposable Gastrointestinal Endoscopic Biopsy Forceps	National Medical Device Registration Approval No. 20253011149	6/20/2025	Gastroenterology & Urology
357	Suzhou Sinofo Medical Technology Co., Ltd.	Digital PCR Analyze	National Medical Device Registration Approval No. 20253221148	6/20/2025	Molecular Genetics
358	Inari Medical，Inc	Pulmonary Artery Thrombectomy System	Imported Medical Device Registration Approval No. 20253030265	6/20/2025	Cardiovascular
359	Sino-European Zhiwei (Shanghai) Robotics Co., Ltd.	Spinal Surgery Navigation and Localization Device	National Medical Device Registration Approval No. 20253011259	6/27/2025	Orthopedic
360	Shenzhen Maiwei Medical Technology Co., Ltd.	Nanosecond Pulsed Field Ablation System	National Medical Device Registration Approval No. 20253011258	6/27/2025	Cardiovascular
361	Syntellix AG	Bioabsorbable Magnesium Alloy Compression Screw	Imported Medical Device Registration Approval No. 20253130280	6/27/2025	Orthopedic
