# Supplementary material for: Comparison of innovative medical devices between China and the United States
Source: Regen Biomater. 2026 Jan 25;13:rbag008. doi: 10.1093/rb/rbag008 (PMC13003204; doi:10.1093/rb/rbag008)
Supplement: rbag008_Supplementary_Data [file rbag008_supplementary_data.zip › Table_S2.pdf]

Table S2. CDRH and CBER Breakthrough Device Marketing Authorizations

NO.	Manufacturer	Trade Name	Marketing Submission Number	Marketing Submission Decision Date	Clinical Panel
1	INSIGHTEC	EXABLATE	P150038	07/11/2016	Neurology
2	FOUNDATION MEDICINE, INC.	FOUNDATIONONE CDX	P170019	11/30/2017	Pathology
3	EMPATICA SRL	EMBRACE	K172935	01/26/2018	Neurology
4	BANYAN BIOMARKERS, INC.	BANYAN BTI	DEN170045	02/14/2018	Neurology
5	CONCENTRIC MEDICAL, INC.	TREVO PRO VUE RETRIEVER AND TREVO XP PRO VUE RETRIEVER (TREVO RETRIEVER)	K173352	02/15/2018	Neurology
6	IDX, LLC	IDX-DR	DEN180001	04/11/2018	Ophthalmology
7	CLINICAL RESEARCH CONSULTANTS, INC.	CUSTOMFLEX ARTIFICIAL IRIS	P170039	05/30/2018	Ophthalmology
8	MEDTRONIC MINIMED, INC.	MINIMED 670G SYSTEM	P160017/S031	06/21/2018	Clinical Chemistry
9	PULMONX CORPORATION	ZEPHYR ENDOBRONCHIAL VALVE SYSTEM	P180002	06/29/2018	Anesthesiology

10	AVITA MEDICAL, LLC.	RECELL AUTOLOGOUS CELL HARVESTING DEVICE	BP170122	9/20/2018	
11	SPIRATION, INC.	SPIRATION VALVE SYSTEM	P180007	12/03/2018	Anesthesiology
12	PEAR THERAPEUTICS	RESET-O	K173681	12/10/2018	Neurology
13	IMPULSE DYNAMICS, INC.	OPTIMIZER SMART SYSTEM	P180036	03/21/2019	Cardiovascular
14	CVRX, INC.	BAROSTIM NEO SYSTEM	P180050	08/16/2019	Cardiovascular
15	ORASURE TECHNOLOGIES	ORAQUICK EBOLA RAPID ANTIGEN TEST	DEN190025	10/10/2019	Microbiology
16	TUSKER MEDICAL	TULA SYSTEM	P190016	11/25/2019	Ear Nose & Throat
17	BOSTON SCIENTIFIC	EXALT MODEL D, SINGLE-USE DUODENOSCOPE, EXALT CONTROLLER	K193202	12/13/2019	Gastroenterology & Urology
18	BAY LABS, INC.	CAPTION GUIDANCE	DEN190040	02/07/2020	Radiology
19	AMBU INNOVATION GMBH	AMBU DUODENO SYSTEM	K201098	07/17/2020	Gastroenterology & Urology
20	ROCHE MOLECULAR SYSTEMS, INC.	COBAS EBV	DEN200015	07/30/2020	Microbiology

21	GUARDANT HEALTH, INC.	GUARDANT360 CDx	P200010	08/7/2020	Pathology
22	FOUNDATION MEDICINE, INC.	FOUNDATIONONE LIQUID CDX	P190032	08/26/2020	Pathology
23	MEDTRONIC MINIMED, INC.	MINIMED 770G SYSTEM	P160017/S076	08/31/2020	Clinical Chemistry
24	ROCHE MOLECULAR SYSTEMS, INC.	COBAS BKV	K202215	09/02/2020	Microbiology
25	FOUNDATION MEDICINE, INC.	FOUNDATIONONE LIQUID CDX	P200006	10/26/2020	Pathology
26	NIGHTWARE, INC.	NIGHTWARE KIT	DEN200033	11/06/2020	Neurology
27	CERUS CORPORATION	INTERCEPT BLOOD SYSTEM FOR PLASMA	BP130076/S034	11/24/2020	
28	CARLSMED, INC.	APREVO INTERVERTEBRAL BODY FUSION DEVICE	K202034	12/03/2020	Orthopedic
29	ABBOTT LABORATORIES	I-STAT ALINITY SYSTEM	K201778	01/08/2021	Immunology
30	SHOCKWAVE MEDICAL, INC.	SHOCKWAVE INTRAVASCULAR LITHOTRIPSY (IVL) SYSTEM WITH SHOCKWAVE C2 CORONARY	P200039	02/12/2021	Committee Cardiovascular
31	MEDTRONIC, INC.	HARMONY TPV SYSTEM	P200046	03/26/2021	Cardiovascular

32	HELIUS MEDICAL, INC.	PORTABLE NEUROMODULATION STIMULATOR (PONS)	DEN200050	03/26/2021	Neurology
33	NEUROLUTIONS, INC.	NEUROLUTIONS IPSIHAND UPPER EXTREMITY REHABILITATION SYSTEM	DEN200046	04/23/2021	Neurology
34	COGNOA, INC.	COGNOA ASD DIAGNOSIS AID	DEN200069	06/02/2021	Neurology
35	CARLSMED, INC.	APREVO TRANSFORAMINAL IBF	K210542	06/30/2021	Orthopedic
36	SIEMENS HEALTHCARE DIAGNOSTICS, INC.	ADVIA CENTAUR ENHANCED LIVER FIBROSIS (ELF)	DEN190056	08/20/2021	Clinical Chemistry
37	CANARY MEDICAL, INC.	CANARY TIBIAL EXTENSION WITH CANARY HEALTH IMPLANTED REPORTING	DEN200064	08/27/2021	Orthopedic
38	MICROTRANSPONDER, INC.	VIVISTIM PAIRED VNS SYSTEM	P210007	08/27/2021	Physical Medicine
39	PAIGE.AI	PAIGE PROSTATE	DEN200080	09/21/2021	Pathology
40	SYNCTHINK, INC.	EYE-SYNC	K202927	10/02/2021	Neurology
41	APPLIEDVR, INC.	EASEVRX	DEN210014	11/16/2021	Neurology
42	KOIOS MEDICAL, INC.	KOIOS DS	K212616	12/16/2021	Radiology

43	SPECTRANETICS, INC.	CAVACLEAR LASER SHEATH	DEN210024	12/21/2021	Cardiovascular
44	INSULET CORPORATION	OMNIPOD 5 AUTOMATED INSULIN DELIVERY SYSTEM	K203768, K203772, AND K203774*	01/27/2022	Clinical Chemistry
45	CARTIHEAL, LTD.	AGILI-C	P210034	03/29/2022	Orthopedic
46	VASCUTEK, LTD.	THORAFLEX HYBRID	P210006	04/19/2022	Cardiovascular
47	ULTRATHERA TECHNOLOGIES, INC.	GYROSTIM	K220231	04/27/2022	Ear Nose & Throat
48	TRANSMEDICS, INC.	ORGAN CARE SYSTEM (OCS) HEART SYSTEM	P180051/S001	04/27/2022	Cardiovascular
49	FUJIREBIO DIAGNOSTICS, INC.	LUMIPULSE G ß-AMYLOID RATIO (1-42/1-40)	DEN200072	05/04/2022	Immunology
50	W. L. GORE & ASSOCIATES, INC.	GORE TAG THORACIC BRANCH ENDOPROSTHESIS (TBE DEVICE)	P210032	05/13/2022	Cardiovascular
51	BONESUPPORT AB	CERAMENT G	DEN210044	05/17/2022	Orthopedic
52	NEUROMETRIX, INC.	QUELL-FM	DEN210046	05/18/2022	Neurology
53	SI-BONE, INC.	IFUSE BEDROCK GRANITE IMPLANT SYSTEM	K220195	05/26/2022	Orthopedic

54	EARLITEC DIAGNOSTICS, INC.	EARLIPOINT SYSTEM	K213882	06/08/2022	Neurology
55	RENOVIA, INC.	LEVA PELVIC HEALTH SYSTEM	K213913	06/30/2022	Gastroenterology & Urology
56	MAGNUS MEDICAL, INC.	MAGNUS NEUROMODULATION SYSTEM (MNS) WITH SAINT TECHNOLOGY, MODEL NUMBER	K220177	09/01/2022	Neurology
57	PHAGENESIS LIMITED	PHAGENYX SYSTEM	DEN220025	09/16/2022	Ear Nose & Throat
58	BRAINCOOL AB	THE COORAL SYSTEM	DEN210027	10/14/2022	Dental
59	ARGENTUM MEDICAL, INC.	SILVERLON WOUND CONTACT, BURN CONTACT DRESSINGS	K221218	10/26/2022	General & Plastic Surgery
60	CARLSMED, INC.	APREVO ANTERIOR LUMBAR INTERBODY FUSION DEVICE WITH INTERFIXATION	K222009	11/22/2022	Orthopedic
61	CALA HEALTH, INC.	CALA KIQ	K222237	11/22/2022	Neurology
62	ULTROMICS LIMITED	ECHOGO HEART FAILURE	K222463	11/23/2022	Cardiovascular
63	ROCHE DIAGONOSTICS	ELECSYS B-AMYLOID (1-42) CSF II, ELECSYS PHOSPHO-TAU (181P) CSF	K221842	12/07/2022	Immunology
64	SI-BONE, INC	IFUSE BEDROCK GRANITE IMPLANT SYSTEM	K222774	12/22/2022	Orthopedic

65	REFLEXION MEDICAL, INC.	REFLEXION MEDICAL RADIOTHERAPY SYSTEM (RMRS)	DEN220014	02/01/2023	Radiology
66	REWALK ROBOTICS, LTD.	REWALK P6.0	K221696	03/02/2023	Neurology
67	ABBOTT LABORATORIES	TBI	K223602	03/02/2023	Immunology
68	BIORETEC, LTD.	REMEOS SCREW LAG SOLID	DEN220030	03/29/2023	Orthopedic
69	MASIMO CORPORATION	MASIMO SAFETYNET OPIOID SYSTEM	DEN200011	03/31/2023	Anesthesiology
70	MOXIMED, INC.	MISHA KNEE SYSTEM	DEN220033	04/10/2023	Orthopedic
71	NOCTRIX HEALTH, INC.	NTX100 TONIC MOTOR ACTIVATION (NTX100 TOMAC) SYSTEM	DEN220059	04/17/2023	Neurology
72	W. L. GORE & ASSOCIATES, INC.	GORE TAG THORACIC BRANCH ENDOPROSTHESIS (TBE DEVICE)	P210032/S007	05/02/2023	Cardiovascular
73	SWING THERAPEUTICS, INC.	STANZA	DEN220083	05/09/2023	Neurology
74	BRAHMS GMBH, PART OF THERMO FISHER SCIENTIFIC	B·R·A·H·M·S SFLT-1/ PLGF KRYPTOR TEST SYSTEM	DEN220027	05/18/2023	Clinical Chemistry
75	CERIBELL, INC.	CERIBELL STATUS EPILEPTICUS MONITOR	K223504	05/23/2023	Neurology

76	ENDOLOGIX, LLC.	DETOUR SYSTEM	P220021	06/07/2023	Cardiovascular
77	AVITA MEDICAL AMERICAS, LLC	RECELL AUTOLOGOUS CELL HARVESTING DEVICE	BP170122/S502	06/07/2023	
78	PREMIA SPINE, LTD.	TOPS SYSTEM	P220002	06/15/2023	Orthopedic
79	AVITA MEDICAL AMERICAS, LLC.	RECELL AUTOLOGOUS CELL HARVESTING DEVICE	BP220799	06/16/2023	
80	ABBOTT MEDICAL	AVEIR DR LEADLESS SYSTEM	P150035/S003	06/29/2023	Cardiovascular
81	RENALYTIX AI, INC.	KIDNEYINTELX.DKD	DEN200052	06/29/2023	Clinical Chemistry
82	BOSTON SCIENTIFIC CORPORATION	VISUAL-ICE CRYOABLATION SYSTEM	K230551	08/08/2023	General & Plastic Surgery
83	ANNALISE-AI PTY, LTD.	ANNALISE ENTERPRISE CTB TRIAGE-OH	K231094	08/15/2023	Radiology
84	LIMACA MEDICAL, LTD.	PRECISION GI	K231422	08/28/2023	Gastroenterology & Urology
85	LIMFLOW, INC.	LIMFLOW SYSTEM	P220025	09/11/2023	Cardiovascular
86	LAMINATE MEDICAL TECHNOLOGIES, LTD.	VASQ	DEN220026	09/26/2023	Cardiovascular

87	ANUMANA, INC.	LOW EJECTION FRACTION AI-ECG ALGORITHM	K232699	09/28/2023	Cardiovascular
88	HISTOSONICS, INC.	EDISON SYSTEM	DEN220087	10/06/2023	General & Plastic Surgery
89	PERFUZE, LTD.	MILLIPEDE 070 ASPIRATION CATHETER, PERFUZE ASPIRATION TUBE SET	K232524	10/18/2023	Neurology
90	RECOR MEDICAL, INC.	PARADISE ULTRASOUND RENAL DENERVATION SYSTEM	P220023	11/07/2023	Cardiovascular
91	MEDTRONIC, INC.	SYMPLICITY SPYRAL RENAL DENERVATION SYSTEM	P220026	11/17/2023	Cardiovascular
92	BIOPORTO DIAGNOSTIC, INC.	PRONEPHRO AKI (NGAL)	K232761	12/07/2023	Clinical Chemistry
93	MEDTRONIC, INC.	PULSESELECT PULSED FIELD ABLATION (PFA) SYSTEM	P230017	12/13/2023	Cardiovascular
94	AUTOGENOMICS, INC.	AVERTD and AVERTD BUCCAL SAMPLE COLLECTION KIT	P230032	12/19/2023	Toxicology
95	ENDOSOUND, INC.	ENDOSOUND VISION SYSTEM	K232518	12/27/2023	Gastroenterology & Urology
96	IMVARIA, INC.	FIBRESOLVE	DEN220040	01/11/2024	Radiology
97	DERMASENSOR, INC.	DERMASENSOR	DEN230008	01/12/2024	General & Plastic Surgery

98	DARMIYAN, INC.	BRAINSEE	DEN220066	01/12/2024	Neurology
99	W.L. GORE & ASSOCIATES, INC.	GORE EXCLUDER THORACOABDOMINAL BRANCH ENDOPROSTHESIS (TAMBE)	P230023	01/12/2024	Cardiovascular
100	BONE HEALTH TECHNOLOGIES, INC.	OSTEOBOOST BELT	DEN230015	01/12/2024	Orthopedic
101	CHEMO MOUTHPIECE, LLC	CHEMO MOUTHPIECE	K232917	01/23/2024	Dental
102	FARAPULSE, INC.	PERCUTANEOUS CARDIAC ABLATION CATHETER FOR TREATMENT OF ATRIAL	P230030	01/30/2024	Cardiovascular
103	EDWARDS LIFESCIENCES, LLC	EDWARDS EVOQUE TRICUSPID VALVE REPLACEMENT SYSTEM	P230013	02/01/2024	Cardiovascular
104	MERIT MEDICAL SYSTEM, INC.	SCOUT MD SURGICAL GUIDANCE SYSTEM	K231468	02/12/2024	General & Plastic Surgery
105	SELUX DIAGNOSTICS, INC.	PBC SEPARATOR	K223493	02/15/2024	Microbiology
106	ICOTEC AG	VADER PEDICLE SYSTEM	K232628	02/26/2024	Orthopedic
107	BOSTON SCIENTIFIC CORPORATION	AGENT PACLITAXEL-COATED BALLOON CATHETER	P230035	02/29/2024	Cardiovascular
108	4WEB MEDICAL, INC.	ANKLE TRUSS SYSTEM (ATS)	K230088	03/21/2024	Orthopedic

109	BONESUPPORT AB	CERAMENT G	K234008	03/24/2024	Orthopedic
110	ABBOTT POINT OF CARE	I-STAT TBI CARTRIDGE WITH THE I-STAT ALINITY SYSTEM	K234143	03/27/2024	Immunology
111	EKO HEALTH, INC.	EKO LOW EJECTION FRACTION TOOL (ELEFT)	K233409	03/28/2024	Cardiovascular
112	INSTANOSIS, INC.	INSTASTRIP FENTANYL RAPID TEST (URINE); INSTASTRIP FENTANYL DIPSTICK TEST	K240295	03/28/2024	Toxicology
113	ABBOTT MEDICAL	TRICLIP G4 SYSTEM	P230007	04/01/2024	Cardiovascular
114	ICOTEC AG	KONG-TL VBR SYSTEM AND KONG C VBR SYSTEM	K232790	04/04/2024	Orthopedic
115	ANALYTICS FOR LIFE, INC.	CORVISTA SYSTEM WITH PH ADD-ON	K233666	04/05/2024	Cardiovascular
116	ICOTEC AG	ICOTEC INTERBODY CAGE SYSTEM (ICOTEC CERVICAL CAGE, ICOTEC PLIF LUMBAR	K232792	04/05/2024	Orthopedic
117	16 BIT, INC.	RHO	DEN230023	04/09/2024	Radiology
118	LUMICELL, INC.	LUMICELL DIRECT VISUALIZATION SYSTEM (DVS)	P230014	04/17/2024	General & Plastic Surgery
119	Q-LINEA AB	ASTAR BC G- KIT AND ASTAR INSTRUMENT	K221688	04/26/2024	Microbiology

120	ABBOTT MEDICAL	ESPRIT BTK EVEROLIMUS ELUTING RESORBABLE SCAFFOLD SYSTEM	P230036	04/26/2024	Cardiovascular
121	GENEOSCOPY, INC.	COLOSENSE	P230001	05/03/2024	Pathology
122	LACTATION LAB, INC.	EMILY'S CARE NOURISH TEST SYSTEM (MODEL 1)	K234088	05/03/2024	Clinical Chemistry
123	NOTAL VISION, INC.	NOTAL VISION HOME OPTICAL COHERENCE TOMOGRAPHY (OCT) SYSTEM	DEN230043	05/15/2024	Ophthalmology
124	ICOTEC AG	ICOTEC ANTERIOR CERVICAL PLATE SYSTEM	K233215	05/17/2024	Orthopedic
125	SPECIFIC DIAGNOSTICS, LLC	VITEK REVEAL GN AST ASSAY AND VITEK REVEAL AST SYSTEM	K230675	06/20/2024	Microbiology
126	ARGENTUM MEDICAL, LLC.	SILVERLON WOUND CONTACT, BURN CONTACT DRESSING	K241225	07/31/2024	General & Plastic Surgery
127	SI-BONE, INC.	IFUSE TORQ TNT IMPLANT SYSTEM	K241504	08/19/2024	Orthopedic
128	NEUROS MEDICAL, INC.	ALTIUS DIRECT ELECTRICAL NERVE STIMULATION SYSTEM	P230020	08/26/2024	Neurology
129	RENATA MEDICAL, INC.	MINIMA STENT SYSTEM	P240003	08/28/2024	Cardiovascular
130	PI-CARDIA, LTD.	SHORTCUT	DEN240017	09/27/2024	Cardiovascular

131	CARLSMED, INC.	APREVO CERVICAL ACDF; APREVO CERVICAL ACDF-X; APREVO® CERVICAL ACDF-X NO	K242260	11/15/2024	Orthopedic
132	ULTROMICS, LTD.	ECHOGO AMYLOIDOSIS (1.0)	K240860	11/15/2024	Cardiovascular
133	MIRUS, LLC.	EUROPA POSTERIOR CERVICAL FUSION SYSTEM	K242516	11/19/2024	Orthopedic
134	GRAVITAS MEDICAL, INC.	ENTARIK NI FEEDING TUBE SYSTEM	K241169	11/22/2024	Gastroenterology & Urology
135	LUNGPACER MEDICAL USA, INC.	AEROPACE SYSTEM	P240012	12/04/2024	Anesthesiology
136	MERIT MEDICAL SYSTEMS, INC.	WRAPSODY CELL-IMPERMEABLE ENDOPROSTHESIS	P240023	12/19/2024	Cardiovascular
137	ONWARD MEDICAL, INC.	ARC-EX SYSTEM	DEN240014	12/19/2024	Physical Medicine
138	IMBIO, INC.	IQ-UIP	K242467	12/19/2024	Radiology
139	SEQUANA MEDICAL NV	ALFAPUMP SYSTEM	P230044	12/20/2024	Gastroenterology & Urology
140	INFLAMMATIX, INC.	TRIVERITY	K241676	01/10/2025	Microbiology
141	IBEX MEDICAL ANALYTICS, LTD.	GALEN SECOND READ	K241232	01/24/2025	Pathology

142	ROCHE DIAGNOSTICS	ELECSYS SFLT-1 AND ELECSYS PLGF	K241453	02/07/2025	Clinical Chemistry
143	MY01, INC.	MY01 CONTINUOUS COMPARTMENTAL PRESSURE MONITOR	K242997	03/13/2025	Orthopedic
144	CARDIOVIA, LTD.	VIAONE EPICARDIAL ACCESS SYSTEM	K243928	03/20/2025	Cardiovascular
145	RESTOR3D, INC.	TIDAL FUSION CAGE SYSTEM	K242356	03/24/2025	Orthopedic
146	SELUX DIAGNOSTICS, INC.	PBC SEPARATOR WITH SELUX AST SYSTEM	K244044	03/28/2025	Microbiology
147	PRAPELA, INC.	PRAPELA SVS HOSPITAL BASSINET PAD (MODEL P01)	DEN240031	04/04/2025	General Hospital
148	ACTIVE PROTECTIVE TECHNOLOGIES, INC.	TANGO BELT (MODEL SAS-001-01 (XS); MODEL SAS-001-02 (S); MODEL SAS-001-03 (M); MODEL	DEN240021	04/09/2025	Physical Medicine
149	EBR SYSTEMS, INC.	WISE (WIRELESS STIMULATION OF THE ENDOCARDIUM TECHNOLOGY) CRT SYSTEM	P240028	04/11/2025	Cardiovascular
150	CLICK THERAPEUTICS, INC.	CT-132	DEN240064	04/11/2025	Neurology
151	EPI-MINDER PTY LTD.	MINDER SYSTEM	DEN240062	04/17/2025	Neurology
152	MEDTRONIC, INC.	OMNIASECURE MRI SURESCAN LEAD MODEL 3930M	P240036	04/22/2025	Cardiovascular

153	TEAL HEALTH, INC.	TEAL WAND	DEN240045	05/09/2025	Microbiology
154	FUJIREBIO DIAGNOSTICS, INC.	LUMIPULSE G PTAU217/ß-AMYLOID 1-42 PLASMA RATIO	K242706	05/16/2025	Immunology
155	INVISION MEDICAL TECHNOLOGY CORPORATION	INVISION PRECISION CARDIAC AMYLOID	K243866	05/21/2025	Cardiovascular
156	RANDOX LABORATORIES LTD.	CONCIZUTRACE ELISA	DEN240035	05/22/2025	Hematology
157	ABBOTT MEDICAL	TENDYNE TRANSCATHETER MITRAL VALVE SYSTEM	P240042	05/23/2025	Cardiovascular
158	REFLOW MEDICAL, INC.	SPUR PERIPHERAL RETRIEVABLE STENT SYSTEM	DEN240048	05/29/2025	Cardiovascular
159	INTUITIVE SURGICAL, INC.	DA VINCI X SURGICAL SYSTEM (IS4200); DA VINCI XI SURGICAL SYSTEM (IS4000)	K240852	06/11/2025	Gastroenterology & Urology
160	CERAPEDICS, INC.	PEARLMATRIX BONE GRAFT	P240001	06/18/2025	Orthopedic
