# Supplementary material for: Comparison of innovative medical devices between China and the United States
Source: Regen Biomater. 2026 Jan 25;13:rbag008. doi: 10.1093/rb/rbag008 (PMC13003204; doi:10.1093/rb/rbag008)
Supplement: rbag008_Supplementary_Data [file rbag008_supplementary_data.zip › supplementary information.docx]

Supplementary Information for

Comparison of innovative medical devices between China and the United States

Xu Song^1,2,3*^, Bo Yuan^2,3^, Kai Zhang^1,2,3^*, Chunying Chen^4,5^, Yuliang Zhao^4,5^, Antonios G. Mikos^6^, Xingdong Zhang^1,2,3^

**The PDF file includes:**

Materials and Methods

Supplementary Text

**Other Supplementary Materials for this manuscript include the following:**

Table S1 to S2

Materials and Methods

Data characteristics and sources

IMDs that obtained granted IMD Designations and marketing authorization in China between October 1, 2014 and June 30, 2025 (table S1) were derived from the designation results of special approval procedure of IMDs published by CMDE of NMPA [7], the data on IMD approvals published by NMPA [8], and NMPA's marketed medical device database [9]. During this period, the data for the U.S. came from data provided by FDA's BDP webpage [9] and Devices@FDA in databases of medical devices [11].

The data on IMDs that obtained granted IMD Designations and marketing authorization

In NMPA, the data (table S1) are organized by Manufacturer, Trade Name, Registration Certificate Number, Registration Certificate Date (first approval), and Clinical Panel. In FDA, the data (table S2) are organized by Manufacturer, Trade Name, Marketing Submission Number, Marketing Submission Decision Date and Clinical Panel.

Analysis of IMD therapeutic categories

The Clinical Panel in table S1 and table S2 is divided according to Device Classification Panels published by FDA [12].

Calculation of CAGR and Percentage of Category

CAGR is calculated as follows:

$$CAGR=\left( \frac{Ending Value}{Beginning Value} \right)^{\frac{1}{number of years}}-1$$

Where: Ending Value and Beginning Value is the final value at the end and the initial value at the start of the period, respectively (The Beginning Value in the CAGR formula cannot be zero). Number of Years is the total number of years over which the growth is measured.

The percentage of each clinical category is calculated as follows:

$Percentage of Category (\%)=\left( \frac{Number of Items in Category}{Total Number of Items} \right)$×100

Where: Number of Items in Category and Total Number of Items refers to the total count of IMDs within that specific clinical category and the overall count of all IMDs across all categories, respectively.

Supplementary Text

Representative IMDs received marketing authorizations in NMPA

Cardiovascular: Fontus^®^ Branched Intraoperative Stent System (Shanghai MicroPort Medical (Group) Co., Ltd.) is a dual-branch segmented release aortic dissection device, suitable for the surgical treatment of Stanford type A and complex Stanford type B aortic dissections that cannot be treated with conventional interventions. Aortic Stent Graft System (Lifetech Scientific (Shenzhen) Co. Ltd.) is an aortic covered stent system suitable for the "chimney technique". It is applicable to patients with Stanford Type B dissection who require revascularization of the left subclavian artery, and the length of the proximal anchoring zone should be ≥ 15 mm. The Transcatheter Aortic Valve System (Jinshi Biotechnology (Changsu) Co., Ltd. ) is applicable to patients who, after being evaluated by the cardiac team, cannot tolerate waiting for the pre-installation time of the conventional transcatheter aortic valve, and who, according to the evaluation by the cardiac team in combination with the scoring system, have symptomatic, calcified, severe degenerative native aortic valve stenosis, are not suitable for conventional surgical valve replacement, and are aged ≥ 70 years. This product is a pre-installed and retrievable transcatheter aortic valve system made by dry-valve technology.

Neurology: Eton® Peripheral Nerve Graft (Jiangsu Yitong Biotechnology Co., Ltd) is designed to repair sensory nerve function of digital nerves, superficial radial nerve branches and forearm median nerve defects within 30 mm in length. NOVA DES® Intracranial Drug-Eluting Stent System (Sino Medical Sciences Technology Inc.) is a healing-oriented drug-eluting stent, suitable for the treatment of symptomatic intracranial atherosclerotic stenosis. NOVA uses eGTM coating technology to promote endothelial cell healing and reduce restenosis. Neurosurgical Planning Software (Sinovation (Beijing) Medical Technology Co., Ltd.) is used for formulating neurosurgical plans. It adopts multi-dimensional spatial vascular reconstruction and avoidance technology, and by combining existing head frame tools and planned paths, it can optimize the surgical path and improve clinical work efficiency.

General & Plastic Surgery: Self-expanding Radioactive Seed-Carried Biliary Stent (Rongsheng (Nanjing) Medical Technology Co., Ltd.) is suitable for the dilation and treatment of bile duct stricture/obstruction caused by malignant tumors that cannot be surgically removed or are unwilling to be surgically removed. It can not only dilate the bile duct stricture, but also deliver radiotherapy the tumor tissue through the radioactive particles carried on the stent. Degradable Magnesium Closing Clip (Suzhou Origin Medical Technology Co., Ltd.) is suitable for the ligation and closure of tubular tissues such as blood vessels or bile ducts that do not require permanent closure force during surgery. It is not suitable for large arteries and veins and can achieve "interventional and non-implantation" treatment. Navigation and Positioning Microwave Ablation System (TrueHealth (Zhuhai) Medical Technology Co., Ltd.) can make a needle insertion plan based on CT images before surgery, and guide the microwave ablation needle for percutaneous puncture surgery during surgery. It is designed for microwave ablation of solid liver tumors in adults.

Ear, Nose, Throat: Dual-Energy Cone Beam Computed Tomography—Ultra3D (LargeV Instrument Corp., Ltd.) is a dual-source, dual-detector oral Cone-Beam Computer Tomography (CBCT) product. The large field of view imaging system is designed for routine imaging examinations of the oral and maxillofacial region, nose and pharyngeal airway. The small field of view imaging system has high spatial resolution and is designed for imaging examinations of the internal structures of the ear (middle ear and inner ear).

Gastroenterology & Urology: Single-port Laparoscope Surgery System (Beijing Surgerii Robotics Company) is designed for laparoscopic surgery in urology and gynecology. The product adopts "deformation drive control technology for continuum structures" to perform surgery in a single-port manner.

Ophthalmic: Multifocal Intraocular Lens (Tianjin Century Healthcare Biomedical Engineering Co., Ltd.) adopts an asymmetric design. It utilizes more levels of diffracted light energy to achieve diversified and free adjustment of diffraction orders. Meanwhile, its phase modulation technology enables personalized design based on individual visual needs, resulting in smooth transition of far, intermediate and near vision and comfortable experience throughout the entire visual range.

Pathology: Microsatellite Instability (MSI) Detection Kit (Real time PCR-Capillary Electrophoresis Method) (Promega (Shanghai) Bioproducts Co., Ltd.) is used to assist in the detection of potential Lynch syndrome in colorectal cancer by qualitatively detecting 8 microsatellite loci in the genomic DNA of tumor tissues from colorectal cancer patients.

Representative BDs received marketing authorizations in FDA

Cardiovascular: Minima Stent System (RENATA MEDICAL, INC.) is a custom-made heart stent for children with congenital heart disease. It is designed to treat pulmonary artery stenosis and coarctation of the aorta in newborns, infants and children weighing at least 1.5 kg and can be expanded periodically over time to keep up with the vascular growth of infants and young children. TriClip™ G4 System (ABBOTT MEDICAL) is a transcatheter, minimally invasive tricuspid valve repair device used to treat tricuspid regurgitation (tricuspid valve leakage) usually caused by cardiac hypertrophy or damage to the tricuspid valve leaflets without open-heart surgery. Edwards EVOQUE Tricuspid Valve Replacement System (EDWARDS LIFESCIENCES, LLC) treats eligible patients with tricuspid regurgitation (TR) through transcatheter valve replacement therapy. ShortCut™(PI-CARDIA, LTD.)is a specialized device designed to separate the leaflets of the existing heart valve. It enables the safe performance of Transcatheter Aortic Valve Replacement (TAVR) in patients with coronary artery occlusion or those at high risk of coronary access-related complications. ECHOGO AMYLOIDOSIS (1.0) (ULTROMICS, LTD.) is a machine learning-based automated decision support system. It assists medical professionals in accurately identifying potential cardiac amyloidosis from complex echocardiographic images when patients aged 65 and above with heart failure undergo routine echocardiographic cardiovascular assessments.

Neurology: Altius Direct Electrical Nerve Stimulation System (NEUROS MEDICAL, INC.) is a patient-controlled, on-demand system that is indicated for the treatment of chronic, refractory phantom pain and residual pain after lower limb amputation in adult amputees by inhibiting pain signaling from damaged peripheral nerves near the amputation site to the central nervous system.

Orthopedic: Agili-C (CARTIHEAL, LTD.) is a cell-free, off-the-shelf implant for cartilage and chondral defects in traumatic and osteoarthritic joints. TOPS System is the only lumbar facet replacement system designed to reduce pressure while maintaining normal motion in patients with lumbar spinal stenosis and degenerative spondylolisthesis, providing superior therapeutic outcomes to lumbar implants used in lumbar fusion surgeries.

General & Plastic Surgery: Lumicell Direct Visualization System (DVS) is a drug and device combination product that provides fluorescent imaging for adjunctive breast cancer tissue detection within the surgical cavity after removal of the primary specimen during lumpectomy.

Clinical Chemistry: MiniMed 770G system (MEDTRONIC MINIMED, INC.) is designed to provide continuous basal rate insulin infusion and insulin bolus infusion for the management of type 1 diabetes in individuals aged two years and above who require insulin; as well as continuous monitoring of glucose level trends in subcutaneous interstitial fluid.

Pathology: ColoSense (GENEOSCOPY, INC.) is a noninvasive colorectal cancer screening test that provides a dynamic view of disease activity by using RNA biomarkers.

Gastroenterology & Urology: Alfapump (SEQUANA MEDICAL NV) is a fully implantable, wirelessly rechargeable device designed for patients with recurrent or refractory ascites due to liver cirrhosis. It can automatically and continuously drain ascites from the abdominal cavity into the bladder.

Hematology: Lumipulse G pTau 217/β-Amyloid 1-42 Plasma Ratio (FUJIREBIO DIAGNOSTICS, INC) is a blood-based in vitro diagnostic products (IVD) used for the early detection of patients aged 55 years and above with Alzheimer's disease (AD)-related amyloid pathology.

Anesthesiology: AeroPace (LUNGPACER MEDICAL USA, INC.) is a transvenous temporary phrenic nerve stimulator, primarily used to help improve diaphragmatic function in patients on mechanical ventilation.
